# An integrated multi-omics analysis of the effects of the food processing-induced contaminant 2-monochloropropane-1,3-diol (2-MCPD) in rat heart

**DOI:** 10.1007/s00204-024-03856-6

**Published:** 2024-09-24

**Authors:** Lucien G. J. Cayer, Thorsten Buhrke, Jennifer Roberts, Andrée Nunnikhoven, Katharina Sommerkorn, Anna Reinhold, Albert Braeuning, Jayadev Raju, Harold M. Aukema, Tobias Karakach

**Affiliations:** 1 Food and Human Nutritional Sciences, University of Manitoba, Winnipeg, MB Canada; 2 Canadian Centre for Agri-Food Research in Health and Medicine, St Boniface Hospital Albrechtsen Research Centre, Winnipeg, MB Canada; 3 Department of Food Safety, German Federal Institute for Risk Assessment, Berlin, Germany; 4 Health Canada, Bureau of Chemical Safety, Ottawa, Canada; 5 Pharmacology, Dalhousie University, Halifax, NS Canada

**Keywords:** 2-MCPD, Cardiotoxicity, Multi-omics, Transcriptome, Proteome, Oxylipin

## Abstract

Many foods including edible oils contain 2-monochloropropane-1,3-diol (2-MCPD), a processing-induced chemical contaminant. Cardiotoxic effects have been shown to result from oral 2-MCPD exposure in rodents, but the underlying mechanisms of action remain poorly understood. We undertook a comprehensive multi-omics approach to assess changes at the transcriptomic, proteomic, and oxylipin levels in heart tissues from male F344 rats that were exposed to 0 or 40 mg/kg BW/day of 2-MCPD in the diet for 90 days, in a regulatory compliant rodent bioassay. Heart tissues were collected for RNA sequencing, quantitative PCR analysis, proteomic analysis via two-dimensional gel electrophoresis and mass spectrometry, and targeted lipidomic profiling by high-performance liquid chromatography–tandem mass spectrometry (HPLC–MS/MS). Transcriptomic and proteomic data analyses revealed upregulation of immune/inflammatory response processes and downregulation of energy metabolism and cardiac structure and functions. Among differentially expressed gene–protein pairs, coronin-1A, a key leukocyte-regulating protein, emerged as markedly up-regulated. Oxylipin profiling highlighted a selective suppression of docosahexaenoic acid-derived metabolites, suggesting a disruption in cardioprotective lipid pathways. These findings suggest that 2-MCPD disrupts homeostasis through inflammatory activation and suppression of metabolic and cardiac function. This research provides insights into 2-MCPD's cardiotoxicity, emphasizing the need for further studies to support hazard characterization.

**Supplementary Information:**

The online version contains supplementary material available at 10.1007/s00204-024-03856-6.

## Introduction

2-Monochloropropane-1,3-diol (2-MCPD) is a processing-induced chemical contaminant of which there is uncertainty concerning the health risks of dietary exposure. Despite the identification of 2-MCPD fatty acid esters in food products that contain refined edible oils (Andres et al. 2013; Schneider et al. 2022; Wöhrlin et al. 2015), there is currently insufficient data for toxicological hazard assessment. The few rodent toxicity studies that have been conducted have identified cardiotoxicity as a primary outcome of 2-MCPD exposure (European Food Safety Authority 2016; Raju et al. 2018, 2016; Schilter et al. 2011). However, the underlying molecular mechanisms of 2-MCPD-induced cardiotoxicity remain to be elucidated.

The majority of toxicological research addressing chloropropanols has focused on 3-monochloropropane-1,2-diol (3-MCPD), as it is typically the most abundant chloropropanol isomer occurring in refined edible oils and foods that contain them (Cui et al. 2021; European Food Safety Authority 2016; Schneider et al. 2022). 3-MCPD has been categorized by the International Agency for Research on Cancer (IARC) as a possible human carcinogen (Group 2B) with the kidneys and testes as primary target organs (IARC 2013). Based on data from animal studies, several food safety authorities have established TDIs for 3-MCPD equivalents ranging from 2 to 4 µg/kg BW/day (Joint FAO/WHO Expert Committee on Food Additive 2017; Knutsen et al. 2018).

Far less is known about 2-MCPD; it has not been evaluated by the IARC, and no toxicity reference values for risk assessment have been established. However, it is formed concomitantly with 3-MCPD at approximately 2:1 (3-MCPD:2-MCPD) in refined oils (Kuhlmann 2016; Tivanello et al. 2020; Wöhrlin et al. 2015), and crucially has unique toxicological activities (Buhrke et al. 2017; Schilter et al. 2011; Schultrich et al. 2017), thus requiring independent evaluation. Previous rodent studies have determined the heart is a primary target organ for 2-MCPD toxicity. Reported effects include non-neoplastic lesion development in the myocardium as well as evidence of elevated oxidative stress response (Buhrke et al. 2018; European Food Safety Authority 2016; Raju et al. 2016, 2018; Schilter et al. 2011). However, the molecular mechanisms underpinning 2-MCPD's impact on cardiac health remain unclear. Thus, there is a need to fill this knowledge gap.

This is particularly significant considering the prevalence of 2-MCPD in foods containing processed oils, notably infant formula. Children under two years were identified as having the highest exposure to 2-MCPD on a per kilogram basis (European Food Safety Authority 2016), representing a potentially vulnerable population. This risk profile, combined with the sparse toxicological data on 2-MCPD, presents a clear need for comprehensive investigation.

In this study, we evaluated the biological processes underlying 2-MCPD-induced cardiotoxicity by analyzing heart tissues from weanling F344 rats exposed to oral 0 and 40 mg/kg body weight (BW)/day 2-MCPD for 90 days; the treatment group was previously shown to have increased heart weights and displayed cardiac lesions characterized by inflammatory cell infiltrate, fibrosis, necrosis, and vacuolation (Supplemental Tables 1–2, Supplemental Fig. 1, Raju et al. 2018). To the best of our knowledge, this study represents the first attempt to measure changes at the transcriptomic, proteomic, and targeted lipidomic levels, a multi-omics approach that captures the complex molecular landscape of 2-MCPD-induced cardiotoxicity. RNA sequencing data provided information regarding the biological processes associated with the gene expression changes. Proteomic data were utilized in determining concomitant protein expression to ascertain the pipeline of gene transcription to protein translation. The lipidomic analysis targeted oxylipins, which are the oxygenated metabolites of polyunsaturated fatty acids (PUFAs), such as arachidonic acid and docosahexaenoic acid (DHA). When PUFA are released from cell membranes by phospholipase A2 (PLA2) activity, oxylipins can be synthesized enzymatically by cytochrome P450 (CYP), cyclooxygenase (COX), or lipoxygenase (LOX) pathways. Oxylipins have vital roles as mediators of cellular processes including inflammation, apoptosis, and fibrosis, and can have variable effects depending on their precursor PUFA (Aukema and Ravandi 2023; Gabbs et al. 2015; Li et al. 2021; Serhan et al. 2015). Additionally, the lipidomic analysis also included oxidized phosphatidylcholines (OxPCs), which are induced by oxidative stress and implicated in atherosclerotic lesions development with roles in inflammation and apoptosis (Allen et al. 2013; Loidl et al. 2003). The enzymes which synthesize oxylipins also contribute to xenobiotic metabolism and different isoforms exhibit varied substrate preferences (Hodgson 2004). The multi-omics integration enabled a comprehensive characterization of the molecular underpinnings of 2-MCPD's cardiotoxicity.

## Materials and methods

### Animals and experimental design

Whole hearts were collected from male F344 rats exposed to dietary 2-MCPD at 0 (control) or 40 mg/kg BW/day (*n* = 6/group). 8-week-old rats received the treatment for 90 days, after which whole hearts were collected, flash-frozen, and pulverized into a homogeneous powder for subsequent molecular analyses. These animals were from the control and the highest dose group of an OECD TG 408-compliant rodent bioassay that provided F344 rats sub-chronic 2-MCPD oral exposure for 90 days, for which 40 mg/kg BW/day was the LOAEL for heart lesions. The 40 mg/kg 2-MCPD dose was selected based on a previous 28-day study which found 50 mg/kg 2-MCPD to be the LOAEL. Body and heart weights, food and water consumption, and heart histopathology results and images can be found in Supplemental Tables 1 and 2 and Supplemental Fig. 1. Additional details of the animal care conditions and toxicological responses from the animals used in this study are described in (Raju et al. 2018).

### Next-generation RNA sequencing

Total RNA was extracted from 25 mg of pulverized frozen and powdered hearts using the miRNEasy RNA extraction kit (Qiagen). RNA-Seq libraries (3′) were prepared from 400 ng of total RNA per sample using the Lexogen QuantSeq 3′-mRNA-Seq Library Prep Kit FWD for Illumina (Greenland, NH, USA) (https://www.lexogen.com/quantseq-3mRNA sequencing/). The resulting libraries were quantified using the Qubit 3.0 Fluorometer—High Sensitivity dsDNA assay (Thermo Fisher Scientific), and validated using the Agilent 2100 Bioanalyzer High Sensitivity DNA chip (Agilent, Mississauga, Canada). Libraries were normalized, pooled and subsequently loaded onto an Illumina NextSeq 500 sequencing system using a NextSeq® 500/550 High Output Kit v2 (75 cycles) flow cell (Illumina, San Diego, CA, USA). After sequencing the libraries, the raw files were pre-processed following standard QC workflows that include demultiplexing, adaptor sequences removal and trimming. These were subsequently mapped to the *Rattus norvegicus* reference genome assembly mRATBN7.2 using STAR, with the –quantMode GeneCounts option which yielded read counts per gene while mapping (Dobin and Gingeras 2015).

### Quantitative PCR

For validation of RNA-seq findings, quantitative polymerase chain reaction (qPCR) was conducted on mRNA extracted from heart tissues. The TaqMan Low Density Array cards (Life Technologies) targeting oxidative stress-related genes were employed. Relative gene expression was calculated using the ΔΔCt method, normalized to 18S rRNA (Schmittgen and Livak 2008).

### Proteomic analysis

The proteomic analysis was performed using methodology previously described in (Sawada et al. 2015, 2016). Briefly, total proteins were extracted from heart tissue and subjected to two-dimensional gel electrophoresis for separation. The gels were stained using Ruthenium II for protein visualization, and images were captured with a VersaDoc Imaging system (Bio-Rad, Munich, Germany). Differentially expressed proteins (DEPs) were identified through spot intensity comparison using ProteinMine software (version 1.6.1, BioImagene). Spots of interest were excised using a SpotXpress HT Spot Picking Robot (Proteome Factory AG, Berlin, Germany), digested with trypsin, and the peptides were analyzed by MALDI-TOF mass spectrometry (MS; Ultraflex II, Bruker). Protein identification was performed against the Mascot database (Perkins et al. 1999), with significance determined by a *P* value < 0.05.

### Targeted lipidomic analysis of oxylipins

The targeted lipidomic analysis data presented here have been reported in (Cayer et al. 2024); the methods used were adapted from (Monirujjaman et al. 2017). Briefly, heart tissues were prepared in Tyrode’s solution with an added mixture of deuterated internal standards. Oxylipins and OxPCs were extracted using solid-phase extraction (Phenomenex, CA, USA) and quantified by high-performance liquid chromatography-tandem MS (Shimadzu Nexera XR coupled to a QTRAP 6500; Sciex, ON, Canada) using a method modified from (Deems et al. 2007) and described in (Aukema et al. 2016; Leng et al. 2017).

### Statistical analyses

All statistical analyses were performed using R (version 4.3.2). RNA-seq and protein expression data were processed using the *edgeR* and *limma* packages to determine differential expression. Briefly, the data were filtered for lowly expressed genes and normalized by the trimmed mean of M values method (Robinson and Oshlack 2010), transformed via voom (Law et al. 2014), and differential expression analysis was determined by an empirical Bayes method (Robinson et al. 2010). Gene set enrichment analysis (GSEA) was performed using the *clusterProfiler* package to identify enriched biological processes and pathways (Subramanian et al. 2005). The GSEA utilized the t scores of all genes included in the differential expression analysis. qPCR and oxylipin data were analyzed by t test when the assumptions of normal distribution and homogeneous variance were met, otherwise values were transformed by Tukey’s power ladder. If assumptions were still not met, the Wilcoxon test was used.

## Results

### Transcriptomic analysis

**Fig. 1:**
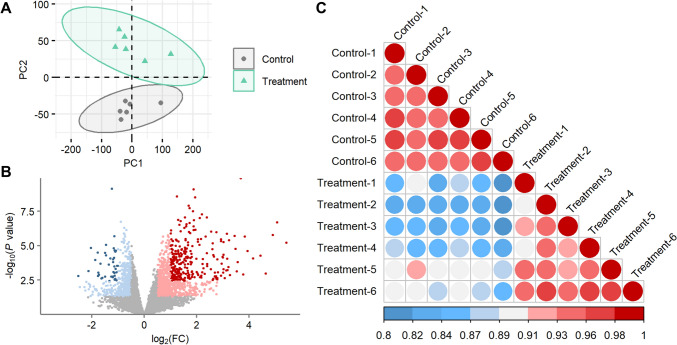
Comparison of rat heart transcriptomic profiles between control and treatment. **a** PCA scores plot of rat heart transcripts. Color overlaid by group. Ellipses illustrate 95% confidence intervals. **b** Volcano plot of rat heart transcripts. Dark blue, LFC < -1 and FDR < 0.05; light blue, LFC < -0.5 and *P* < 0.05; gray, no change; pink, LFC > 0.5 and *P* < 0.05; red, LFC > 1 and FDR < 0.05. **c** Correlation plot of sample DEG profiles. Colors represent Pearson correlation coefficients

To evaluate the impact of 2-MCPD on the cardiac transcriptome, we conducted a comprehensive RNA-seq analysis on rat hearts from 2-MCPD-treated animals. After removal of lowly expressed genes, 10,853 genes were included in the analysis and displayed distinct transcriptomic profiles between the control and 2-MCPD-treated group, when modeled by principal component analysis (PCA) (Fig. 1a). Differential gene expression analysis, with differentially expressed genes (DEGs) defined as |LFC|> 1 and FDR < 0.05, revealed more upregulation (300 genes) than downregulation (41 genes) (Fig. 1b, listed in Supplemental Table 3). Higher Pearson correlation coefficients between sample DEG expression profiles from the same group (*r* = 0.90–0.96) than between groups (*r* = 0.80–0.92) confirmed the consistency and reliability of these expression patterns (Fig. 1c). These dysregulations highlight a considerable transcription level response to 2-MCPD exposure.

**Fig. 2:**
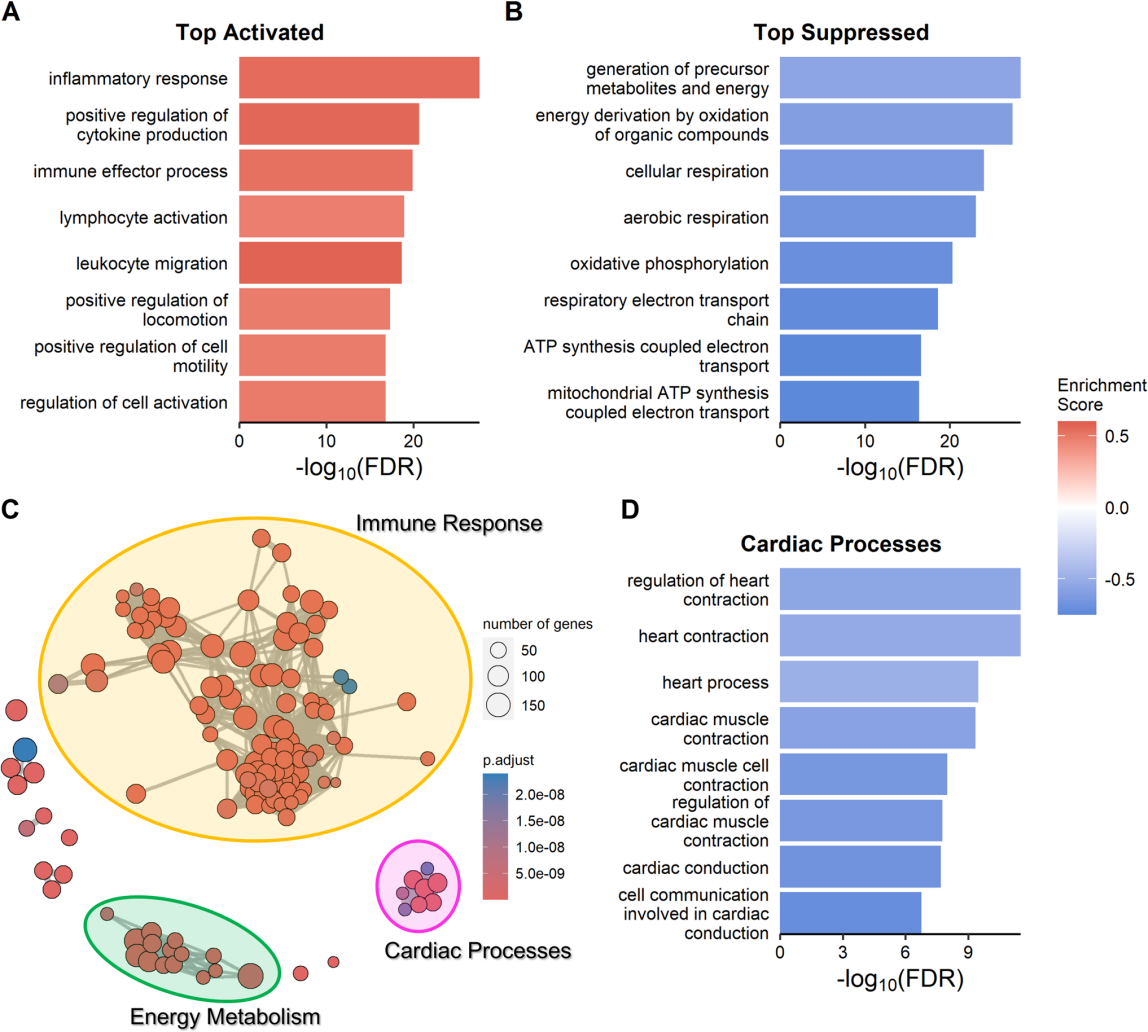
Gene set enrichment analysis of Gene Ontology biological processes. **a** Positively enriched GO terms by top FDR. **b** Negatively enriched GO terms by top FDR. **c** Network of enriched processes with nodes as GO terms. Edge length inversely related to the number of similar genes in the connected sets. **d** Enriched cardiac processes by FDR. All processes altered were suppressed

GSEA using the Gene Ontology (GO) database provided insights into the biological processes most associated with the gene expression changes in response to 2-MCPD exposure. The positively enriched (activated) GO terms were predominantly related to immune response processes, with the top 49 terms by FDR exclusively immunological, demonstrating the significant extent to which immune response processes are up-regulated (Fig. 2a, Supplemental Table 4a). The analysis also identified decreased mitochondrial function as gene sets related to energy metabolism-related processes were most negatively enriched (suppressed) (Fig. 2b, Supplemental Table 4b). A network analysis of this GSEA result revealed three clusters (Fig. 2c). The largest cluster included the immune response-related processes, further emphasizing the inflammatory impact of 2-MCPD on cardiac tissue. Another cluster comprised energy metabolism-related processes, and the third contained cardiac processes. The significantly altered cardiac processes were all suppressed indicating decreased cardiovascular function (Fig. 2d, Supplemental Table 4b). From the histopathology results reported in (Raju et al. 2018), we also had interest in fibrotic processes, and identified 7 enriched processes related to fibroblast migration and proliferation (Supplemental Table 5). GSEA using the Kyoto Encyclopedia of Genes and Genomes (KEGG) corroborated these findings, illustrating activation of numerous immune pathways, particularly those involving leukocyte functions, such as chemokine signaling, cytotoxicity, phagocytosis, and migration, alongside signaling processes in apoptosis, inflammation, and cell differentiation. Conversely, energy metabolism pathways, including oxidative phosphorylation and propanoate metabolism, along with the cardiac muscle contraction pathway, were identified as downregulated (Supplemental Figs. 2–16).

### Quantitative PCR panel

Investigation into oxidative stress responses and validation of the RNA-seq data was conducted via a qPCR assay. Six out of the 91 genes measured in the oxidative stress panel (*Actb, Apoe, Ncf2, Ptgs2, Srxn1, Txnrd1*) were considered DEGs (|LFC|> 1 and FDR < 0.05), all of which were up-regulated from control values, suggesting some increase to oxidative stress response (Supplemental Table 6). Notably, *Ptgs2* emerged as the most up-regulated gene in the panel. As the gene encoding COX-2, the rate-limiting enzyme in prostaglandin synthesis, *Ptgs2* is strongly associated with inflammation and linked to cardiac fibrosis, conditions associated with chronic oxidative stress (Aragno et al. 2008; Li et al. 2021; Maulik and Kumar 2012; Ricciotti and Fitzgerald 2011).

Importantly, the RNA-seq data were in good concordance with the qPCR results. The set of matched qPCR and RNA-seq genes with FDR < 0.05 from either analysis is displayed in Table 1. It revealed all genes, including *Ptgs2*, to have LFCs in the same direction, except for *Atr* which had *P* > 0.05 in the RNA-seq analysis.

**Table 1:** Comparison of qPCR array and RNA-seq results on genes with FDR < 0.05 in either analysis, sorted by qPCR LFC

	qPCR		RNA-seq	
Gene	LFC	*P* value	LFC	*P* value
*Ptgs2*	3.24	0.002	2.37	0.007
*Actb*	1.22	< 0.001	0.902	< 0.001
*Apoe*	1.03	< 0.001	0.791	0.005
*Txnrd1*	1.02	0.002	0.813	0.003
*Gpx7*	0.831	< 0.001	0.393	0.068
*Gpx8*	0.814	< 0.001	0.928	< 0.001
*Gusb*	0.768	0.004	0.593	0.020
*Prdx4*	0.764	< 0.001	0.494	0.033
*Cygb*	0.735	< 0.001	0.561	0.017
*Ctsb*	0.670	0.001	0.597	< 0.001
*Vim*	0.662	0.011	0.687	< 0.001
*Atr*	0.625	0.002	− 0.207	0.537
*Ptgs1*	0.581	0.005	0.496	0.043
*Nox4*	0.574	< 0.001	0.058	0.843
*Ppp1r15b*	0.524	0.002	0.239	0.510
*Rplp0*	0.502	0.009	0.349	0.001
*Gsr*	0.452	0.004	0.00	0.999
*Idh1*	0.438	0.003	0.259	0.136
*Aqr*	0.322	0.010	0.117	0.631
*Gstk1*	− 0.262	0.086	− 0.597	0.003
*Slc41a3*	− 0.287	0.175	− 0.536	< 0.001
*Ucp3*	− 0.412	0.057	− 0.704	< 0.001

### Proteomic analysis

**Fig. 3:**
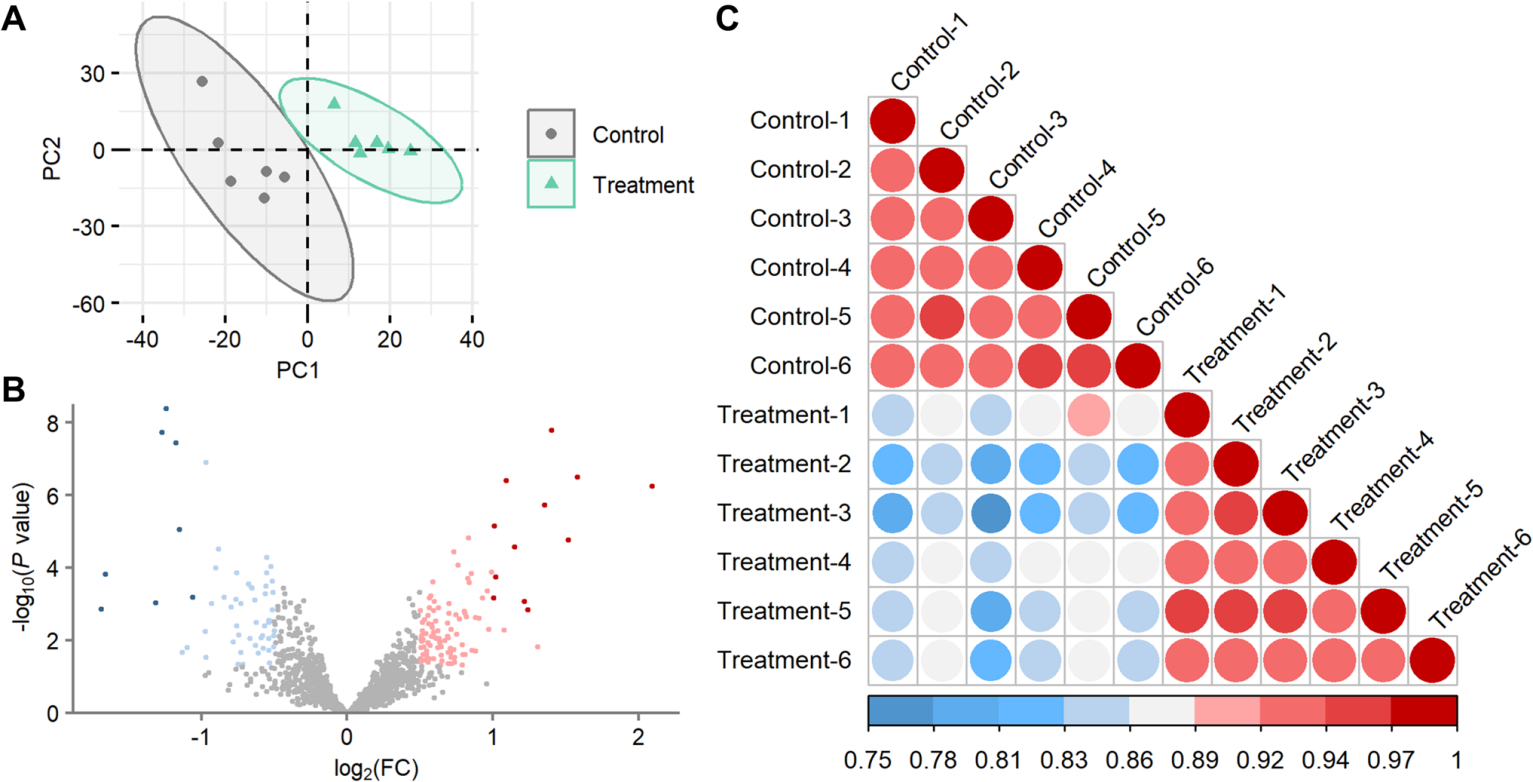
Comparison of rat heart proteomic profiles between control and treatment. **a** PCA scores plot of rat heart proteins. Color overlaid by group. Ellipses illustrate 95% confidence intervals. **b** Volcano plot of rat heart proteins. Dark blue, LFC < -1 and FDR < 0.05; light blue, LFC < -0.5 and *P* < 0.05; gray, no change; pink, LFC > 0.5 and *P* < 0.05; red, LFC > 1 and FDR < 0.05. **c** Correlation plot of sample DEG profiles. Colors represent Pearson correlation coefficients

A proteomic analysis was also conducted to examine the impact of 2-MCPD in rat heart at protein level. The proteomic profiles included 1341 protein spot intensities. A PCA model which included all measured proteins revealed distinct groupings of the control and treatment groups (Fig. 3a). Differential expression analysis identified 20 DEPs, with 8 downregulated and 12 up-regulated, which were induced by 2-MCPD treatment (Fig. 3b). Stronger correlations (*r* = 0.92–0.96) between the DEP expression values of samples from the same group than between groups (*r* = 0.76–0.89) indicated reliable data and consistent treatment effects occurred (Fig. 3c).

**Fig. 4:**
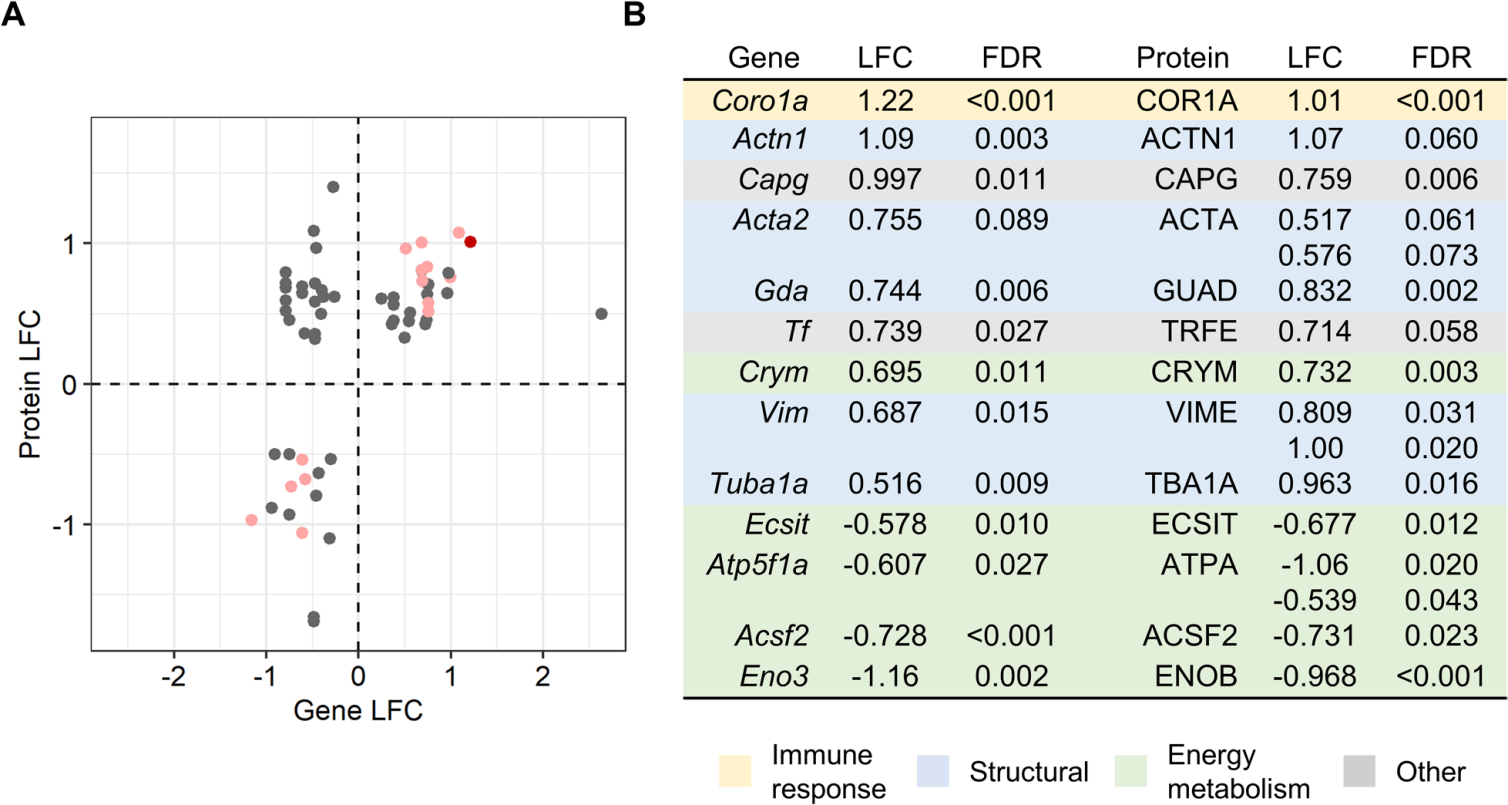
Matched rat heart gene–protein pairs differentially expressed with 2-MCPD treatment. **a** Scatter plot of matched significantly altered (*P* < 0.05) genes and proteins by expression in LFC. Red, gene and protein expression |LFC|> 1 and FDR < 0.05; pink, gene and protein expression |LFC|> 0.5 and FDR < 0.1; gray, gene and/or protein not differentially expressed. **b** DEG-DEP expression values

Of the protein spot intensities measured, a subset of 203 were identified by MS and are listed in Supplemental Table 7. The identified proteins were matched with their corresponding genes from the RNA-seq analysis and plotted by expression when both gene and protein were significantly altered (*P* < 0.05) in Fig. 4a. The DEG–DEP pair *Coro1a*-COR1A was concomitantly up-regulated with the strictest cutoffs of |LFC|> 1 and FDR < 0.05, thus identifying the corresponding coronin-1A as a protein of particular interest in 2-MCPD exposure. An additional 4 downregulated (ACSF2, ATPA, ECSIT, and ENOB) and 9 up-regulated (ACTA, ACTN1, CAPG, CRYM, GUAD, TBA1A, TRFE, and VIME) gene–protein pairs of interest were identified when the correlation analysis was expanded to include expression of |LFC|> 0.5 and FDR < 0.1 (Fig. 4b). The downregulated gene–protein pairs were each involved in energy metabolism, whereas the up-regulated gene–protein pairs were predominantly related to inflammation or structural proteins.

### Differential expression of oxylipin metabolism genes

The oxylipins of the 2-MCPD-exposed rat heart were of interest due to the dysregulation of oxylipin production genes. This particularly accounts for *Ptgs2* (COX-2) due to its function in the rate-limiting step of PG synthesis and its association with cardiac fibrosis and inflammation (Li et al. 2021; Ricciotti and Fitzgerald 2011). The mRNA expression data were filtered for oxylipin metabolism-related genes, of which 13 genes exhibited differential expression of |LFC|> 0.5 and *P* < 0.05 (Table 2, for full list see Supplemental Table 8). In addition to *Ptgs2*, two other genes (*Ptgis* and *Txbas1*) from the COX pathway were up-regulated, suggesting prostanoid synthesis would be up-regulated. Three CYP genes were altered, two downregulated (*Cyp2e1* and *Cyp4b1*) and one up-regulated (*Cyp1b1*), suggesting expected decreases in the epoxy-PUFA, dihydroxy-PUFA, and hydroxy-PUFA produced by the corresponding CYP enzymes. Additionally, soluble epoxide hydrolase (*Ephx2*) was downregulated, suggesting suppressed conversion of epoxy-PUFA to dihydroxy-PUFA. Three PLA2 genes involved in the liberation of PUFA from the cell membrane were altered with two downregulated (*Pla2g2a* and *Pla2g5*) and one up-regulated (*Pla2g2d*). Each of these genes belongs to the secreted PLA2 (sPLA2) subfamily, which have selective substrate preference for DHA (Manson et al. 2023). Thus, a general decrease in DHA oxylipins could be expected. From the proteomic analysis, glutathione peroxidase 1 was the only oxylipin-metabolizing enzyme identified, and it was not significantly altered (*P* = 0.212).

**Table 2:** Oxylipin metabolism DEGs from RNA-seq analysis of rat heart

Pathway	Gene	LFC	*P* value
COX	*Ptgis*	0.737	0.014
	*Ptgs2*	2.37	0.007
	*Tbxas1*	0.761	0.040
CYP	*Cyp1b1*	1.95	< 0.001
	*Cyp2e1*	− 1.77	< 0.001
	*Cyp4b1*	− 0.918	< 0.001
	*Ephx2*	− 0.528	0.012
LOX	*Gpx8*	0.928	< 0.001
	*Ltc4s*	− 0.961	0.014
PLA2	*Pla2g2a*	− 0.759	0.019
	*Pla2g2d*	0.760	0.006
	*Pla2g5*	− 0.959	0.002

### Targeted lipidomic analysis

**Fig. 5:**
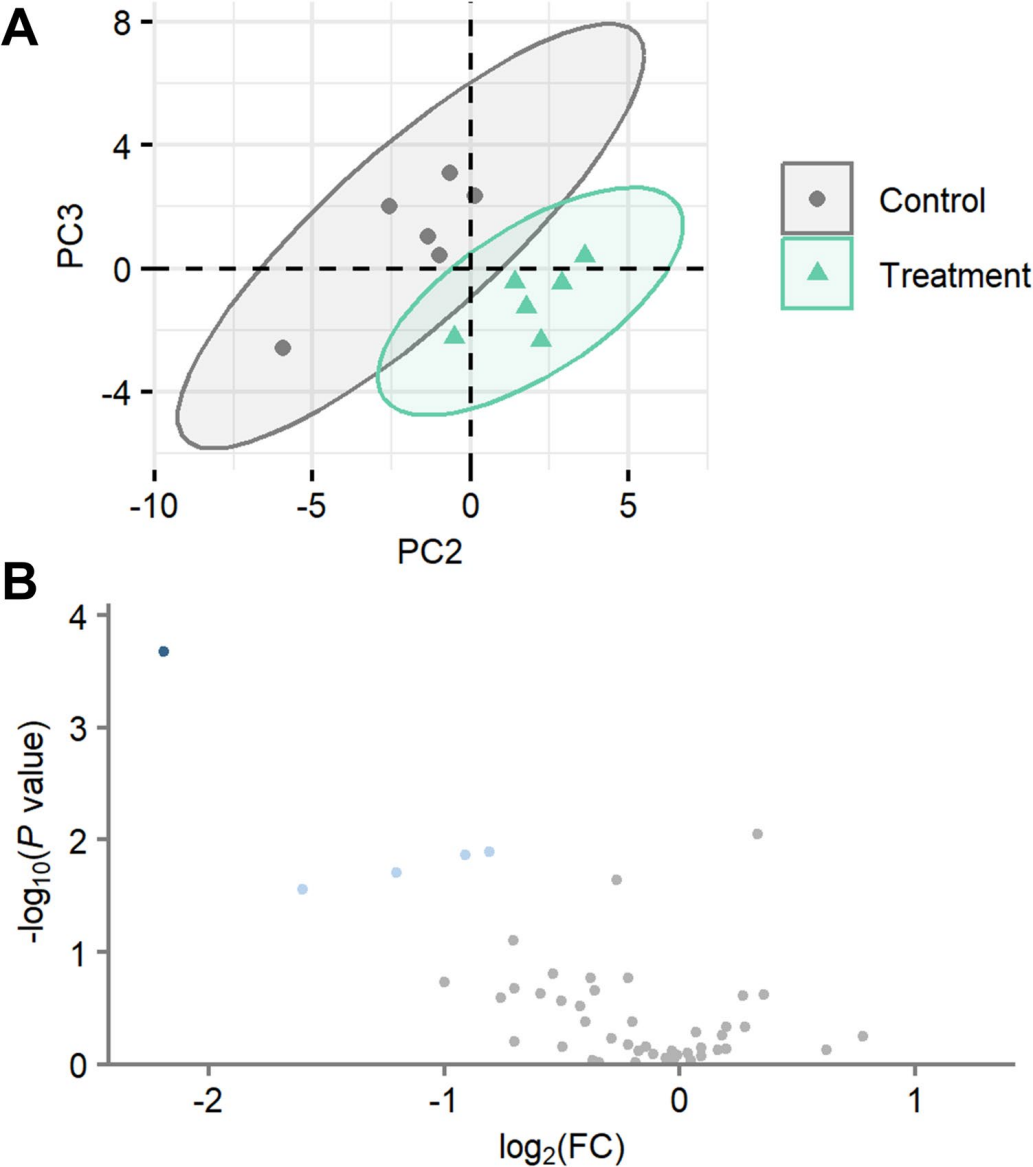
Comparison of rat heart oxylipin profiles between control and treatment. **a** PCA scores plot of rat heart oxylipins. Color overlaid by group. Ellipses illustrate 95% confidence intervals. **b** Volcano plot comparison of rat heart oxylipins between control and treatment. Dark blue, LFC < -1 and FDR < 0.05; light blue, LFC < − 0.5 and *P* < 0.05; gray, no change

**Fig. 6:**
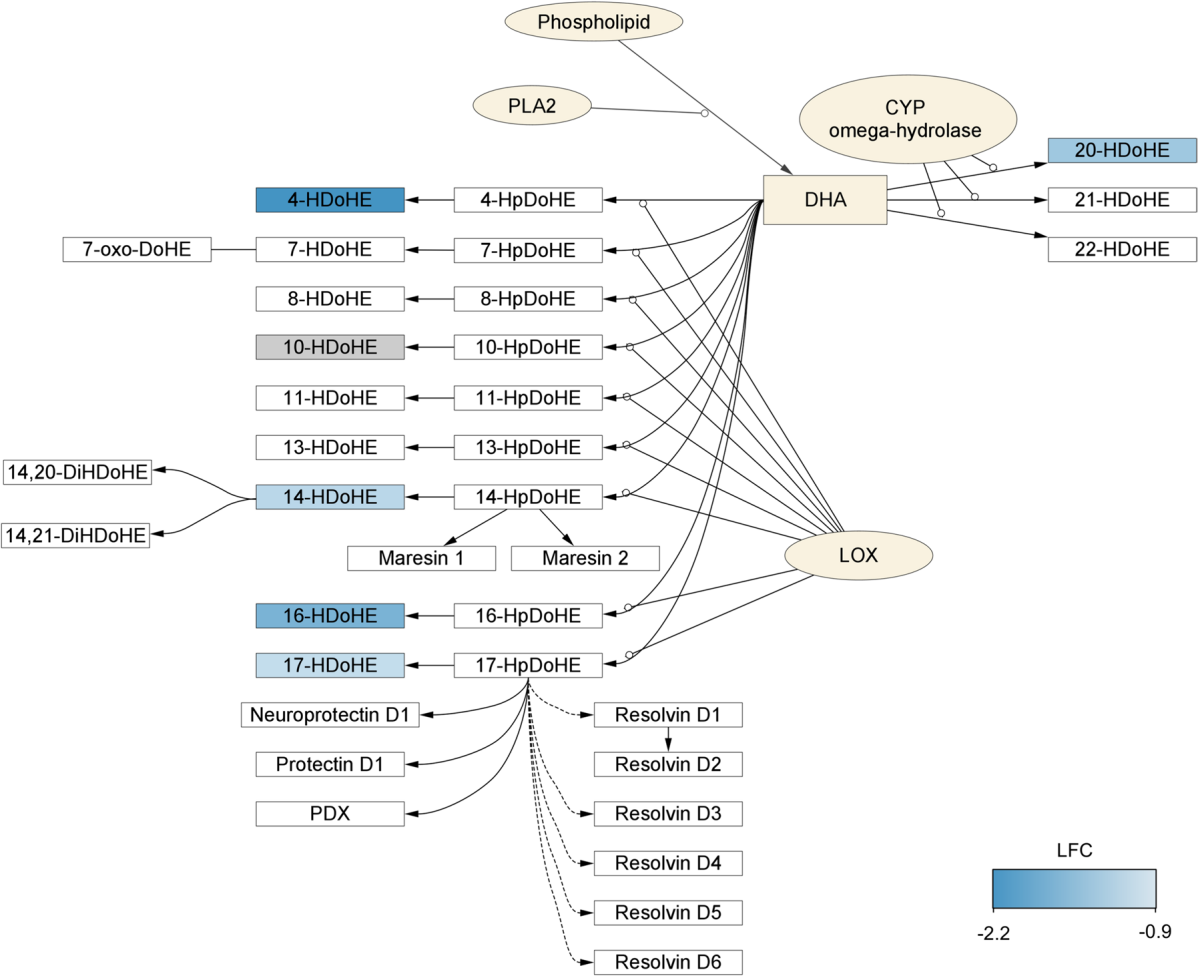
DHA oxylipin synthesis via LOX and CYP ω-hydrolase pathways. Quantified oxylipins colored in shades of blue if differentially expressed or gray if not altered. White, Oxylipins < LOQ; khaki, enzymes and non-oxylipin lipids

Despite the alterations in expression of genes regulating various oxylipin producing pathways, the oxylipin analysis revealed a specific effect of suppression of DHA-derived oxylipins from both LOX and CYP pathways. Of the 53 oxylipins quantified, 5 were differentially expressed (|LFC|> 0.5 and *P* < 0.05; Fig. 5), all 5 of which were DHA metabolites (Table 3 and Supplemental Table 9). In total, 6 DHA oxylipins were quantified, thus 5/6 were differentially expressed (Fig. 6), and the total DHA oxylipin mass was reduced (*P* = 0.002, Supplemental Table 9). Notably, DHA and its metabolites are associated with cardioprotective properties, shedding light on the molecular perturbance of 2-MCPD treatment.

**Table 3:** List of differentially expressed oxylipins in the male rat heart exposed to 2-MCPD for 90 days

Precursor	Enzyme pathway	Oxylipin	LFC	*P* value	FDR
DHA	LOX	4-HDoHE	− 2.19	< 0.001	0.011
	LOX	14-HDoHE	− 0.909	0.014	0.181
	LOX	16-HDoHE	− 1.60	0.028	0.210
	LOX	17-HDoHE	− 0.806	0.013	0.181
	CYP	20-HDoHE	− 1.20	0.019	0.203

OxPCs were also of interest, particularly as markers of oxidative stress. Of the 3 OxPCs measured, none were significantly altered (*P* < 0.05), indicating oxidative stress did not influence oxidized lipid expression between groups (Supplemental Table 10).

## Discussion

This study elucidated molecular processes underlying cardiotoxic effects of 2-MCPD exposure in male F344 rats, by integrating the alterations in transcriptomic, proteomic, and targeted lipidomic profiles. Our findings revealed a multi-omic disruption of cardiac homeostatic processes, characterized by activated immune responses and suppression of mitochondrial function and associated energy production, as well as suppressed cardiac muscle contraction and conduction. Transcriptomic analysis highlighted a pronounced response to 2-MCPD exposure, with immune response genes markedly up-regulated, indicating an active inflammatory state within the heart tissue. Concurrently, downregulated genes involved in energy metabolism and cardiac function reveal 2-MCPD disrupted cardiac homeostasis. Proteomic data corroborated these findings, revealing differential expression of proteins related to immune response, energy metabolism, and cardiac structure, thereby illustrating that the 2-MCPD-induced perturbation of heart tissue went beyond the transcriptome. The targeted lipidomic analysis further elucidated the proinflammatory conditions within the heart tissue by identifying decreased levels of anti-inflammatory DHA-derived oxylipins (Bento et al. 2011; Croset et al. 1988; González‐Périz et al. 2006; Weylandt et al. 2011). Additionally, the absence of significant alterations in OxPCs and limited upregulation of oxidative stress response genes indicate oxidative stress did not play a predominant role in 2-MCPD-induced cardiotoxicity. Overall, the molecular alterations implicate both inflammation and energy deprivation as mechanisms central to the cardiotoxicity of 2-MCPD in rats.

The data presented here are well-supported by the histopathological changes reported in (Raju et al. 2018), which included lesion development of the myocardium and cardiac interstitium characterized by inflammatory cell infiltrate, fibrosis, necrosis, and vacuolation. Our findings also provide useful insights into the underlying processes which occurred. We characterize the immune response through a series of activated processes, pathways, and individual molecules. For instance, the identification of coronin-1A (gene and protein) upregulation further enhances the understanding of the detrimental inflammation. Coronin-1A is an actin-binding protein that is involved in leukocyte adhesion, phagocytosis, and persistence (Moriceau et al. 2009; Pick et al. 2017; Siegmund et al. 2015; Yan et al. 2005). Upregulation of coronin-1A may have inhibited inflammation resolution and should be a target for future study. The activation of fibroblast-related processes comports with the observed cardiac fibrosis. Furthermore, fibrotic cardiac tissue decreases contractility, corresponding to the molecular results (Frangogiannis 2021). The suppression of DHA-derived oxylipins, which are known for their inflammation-resolving properties (Gabbs et al. 2015), suggests that 2-MCPD exposure may compromise the heart's protective mechanisms against inflammation, contributing to the observed cardiotoxicity. We can also postulate that decreased energy metabolism could contribute to necrotic cell death, which in turn promotes inflammation (Festjens et al. 2006; Koo et al. 2015). Further work is needed to determine this causal chain. Overall, the results suggest that 2-MCPD exposure disrupts cardiac homeostasis by triggering a persistent inflammatory state and decreasing energy metabolism, leading to cardiac lesion development and decreased heart contractility. There are no data available on alterations to the heart rate of 2-MCPD-treated rats, however, it must be noted that the heart weights of the 2-MCPD-treated animals were significantly increased compared to those of the control animals (Raju et al. 2018), which suggests an adaptive compensation to maintain cardiac output.

The suppression of DHA-derived oxylipins was congruent with the overall downregulation of the PLA2 genes measured because the altered genes (*Pla2g2a*, *Pla2g2d*, and *Pla2g5*) are sPLA2 isoforms, which have selective preference for DHA and regulate the majority of downstream DHA oxylipin production (Hernandez-Anzaldo et al. 2015; Manson et al. 2023). *Pla2g5*, the most differentially expressed of the PLA2 genes, is implicated in regulating cardiac inflammation via oxylipin manipulation (Berry et al. 2015). In addition to the anti-inflammatory functions of the DHA oxylipins measured (Bento et al. 2011; Croset et al. 1988; González‐Périz et al. 2006; Weylandt et al. 2011), the reduced levels of 14-HDoHE and 17-HDoHE suggest a reduction in the synthesis of the potent anti-inflammatory, pro-resolving, and anti-fibrotic maresins, protectins, and resolvins, because they are derived from common hydroperoxyl-DHA precursors (Fig. 6) (Deng et al. 2014; Gabbs et al. 2015; Lagarde et al. 2020; Serhan 2014). Thus, the suppression of DHA oxylipins and their biosynthetic pathways explicate the effects of 2-MCPD on cardiac inflammation via lipid mediators. Additionally, the evidence of dietary n-3 PUFAs to modulate oxylipins suggests a role for n-3 PUFA supplementation to mitigate the adverse effects of 2-MCPD (Ferdouse et al. 2019).

The oxylipin analysis also further emphasized the importance of a multi-omics approach in revealing the complex molecular effects of 2-MCPD exposure. The lack of alterations to oxylipins from PUFA precursors excluding DHA, despite the differential expression of oxylipin synthesis genes, such as *Ptgs2*, exemplified the issues with predicting biological effects based solely on genetic analyses. Namely, that metabolite expression does not necessarily reflect changes at the genetic level.

Previously, oxidative stress had been postulated as a molecular contributor to 2-MCPD-induced toxicity (Buhrke et al. 2018). Here we identified a sparse set of oxidative stress response-related genes in the heart up-regulated with 2-MCPD exposure, while OxPCs, lipid markers of oxidative stress, were not altered. Furthermore, the GSEA did not identify oxidative stress response activation, and instead revealed decreased metabolic processes, such as oxidative phosphorylation, which would theoretically produce less oxidative stress (Kowalczyk et al. 2021). Additionally, oxidative stress analyses conducted in our study demonstrated an absence of alterations in the systemic oxidative stress markers: 4-HNE, 8-OHdG, nitrotyrosine, reported in (Raju et al. 2018); and in kidney, muscle, and serum level oxidative stress markers: isoprostanes and OxPCs, reported in (Cayer et al. 2024). Similarly, mice given a 28-day exposure of 2-MCPD showed limited evidence of oxidative stress in the kidney and testes only (Schultrich et al. 2020). Taking all this into account, oxidative stress response was not likely to be a primary driver of the cardiotoxic effects observed.

The implications of our findings for public health remain to be determined, but considering the widespread presence of 2-MCPD in processed foods and that the highest per BW consumers are children under 2 years old (European Food Safety Authority 2016), determining the impact to humans should remain a priority. While our study provides crucial insights into the cardiotoxic mode-of-action of 2-MCPD, the translation of these findings from animals to humans requires careful consideration. The 40 mg/kg BW/day dose used in this study is over 4 orders of magnitude greater than the estimated high human intake of 1 µg/kg BW/day (European Food Safety Authority 2016). Furthermore, virtually no effects were observed in the next lowest dose group of 10 mg/kg BW/day reported in (Raju et al. 2018). Therefore, it is unlikely that dietary exposure to 2-MCPD alone would have comparable impacts for human heart health. It should, however, be noted that the duration of human exposure is much longer, and chronic sub-toxic exposure levels should be taken into consideration. Moreover, there are almost no data available regarding the absorption, distribution, metabolism, and excretion of 2-MCPD in the human body. These bioavailability data, however, are required to characterize the toxic potential of this substance. Finally, there is also the potential of a toxic mixture effect with the isomeric substances 3-MCPD and glycidol, as 2-MCPD occurs concomitantly with these other contaminants in processed food oils (Nguyen and Fromberg 2020; Wöhrlin et al. 2015). A robust understanding of the impacts of 2-MCPD, as contributed to by this study, is however a prerequisite of that work. Nonetheless, our study supports the importance of monitoring 2-MCPD levels in food products and the need for further research into its health impacts.

## Conclusions

Our study examined the multifaceted impact of 2-MCPD on cardiac tissue at the molecular level, implicating inflammatory responses, suppressed energy metabolism, and impaired cardiac function. These findings highlight the complexity of 2-MCPD's cardiotoxic mode-of-action and build upon our understanding of the biological processes underpinning it, in a manner consistent with the histopathological findings. Ultimately, this study supports the necessity for comprehensive risk assessments of 2-MCPD as a food contaminant.

## Supplementary Information

Below is the link to the electronic supplementary material.

Supplementary file1 (DOCX 1848 KB)

Supplementary file2 (DOCX 109 KB)

## Data Availability

Data will be made available upon request.

## Abbreviations

2-MCPD: 2-Monochloropropane-1,3-diol
3-MCPD: 3-Monochloropropane-1,2-diol
BW: Body weight
COX: Cyclooxygenase
CYP: Cytochrome P450
DEG: Differentially expressed gene
DEP: Differentially expressed protein
DHA: Docosahexaenoic acid
FDR: Benjamini–Hochberg-adjusted *P* value, GO, Gene Ontology
GSEA: Gene set enrichment analysis
KEGG: Kyoto Encyclopedia of Genes and Genomes
LFC: Log_2_(fold change)
LOX: Lipoxygenase
MS: Mass spectrometry
OxPC: Oxidized phosphatidylcholine
PCA: Principal component analysis
PLA2: Phospholipase A2
PUFA: Polyunsaturated fatty acid
qPCR: Quantitative polymerase chain reaction
RNA-seq: Next-generation RNA sequencing
