# Supplementary material for: An integrated multi-omics analysis of the effects of the food processing-induced contaminant 2-monochloropropane-1,3-diol (2-MCPD) in rat heart
Source: Arch Toxicol. 2024 Sep 24;98(12):4033–45. doi: 10.1007/s00204-024-03856-6 (PMC11496350; doi:10.1007/s00204-024-03856-6)

# Supplemental Figures.

# Supplemental Figure 1. Histopathology slide from heart tissue (left ventricle) of rats in the 90-day study of oral 2-MCPD exposure.

Significant increases in non-neoplastic lesions were observed in the hearts of 40 mg/kg BW 2-MCPD-treated rats. Lesions were characterized as multifocal myocardial and interstitial vacuolation (arrows) and necrosis with inflammatory cell infiltrate (arrowheads). H7E stain, 20x.

A) Male Control B) Male 40 mg/kg


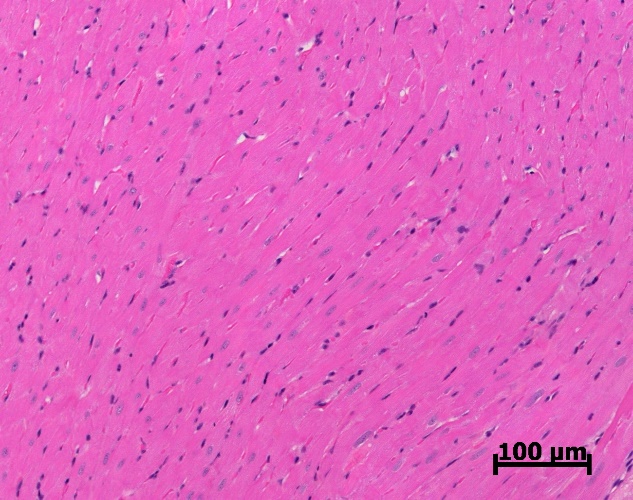

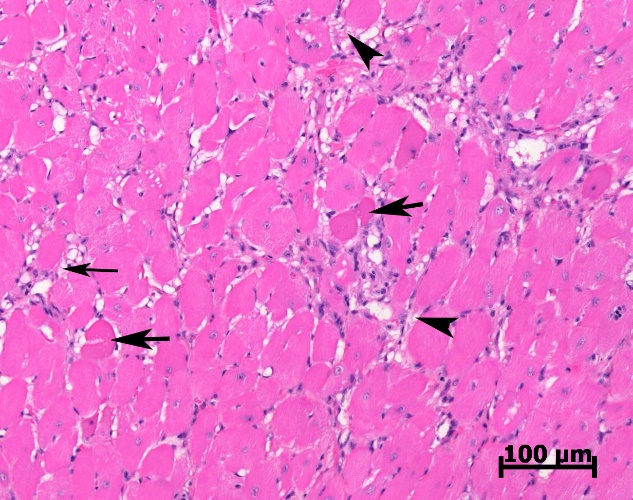


## Gene set enrichment analysis of 2-MCPD-exposed F344 rat heart mRNA using Kyoto Encyclopedia if Genes and Genomes (KEGG) pathways.

Significantly enriched KEGG pathways. DEGs (|LFC| > 0.5 & *P* < 0.05) coloured green when downregulated and red when upregulated.

## Immune Response

#### *rno04062* “Chemokine signalling pathway”


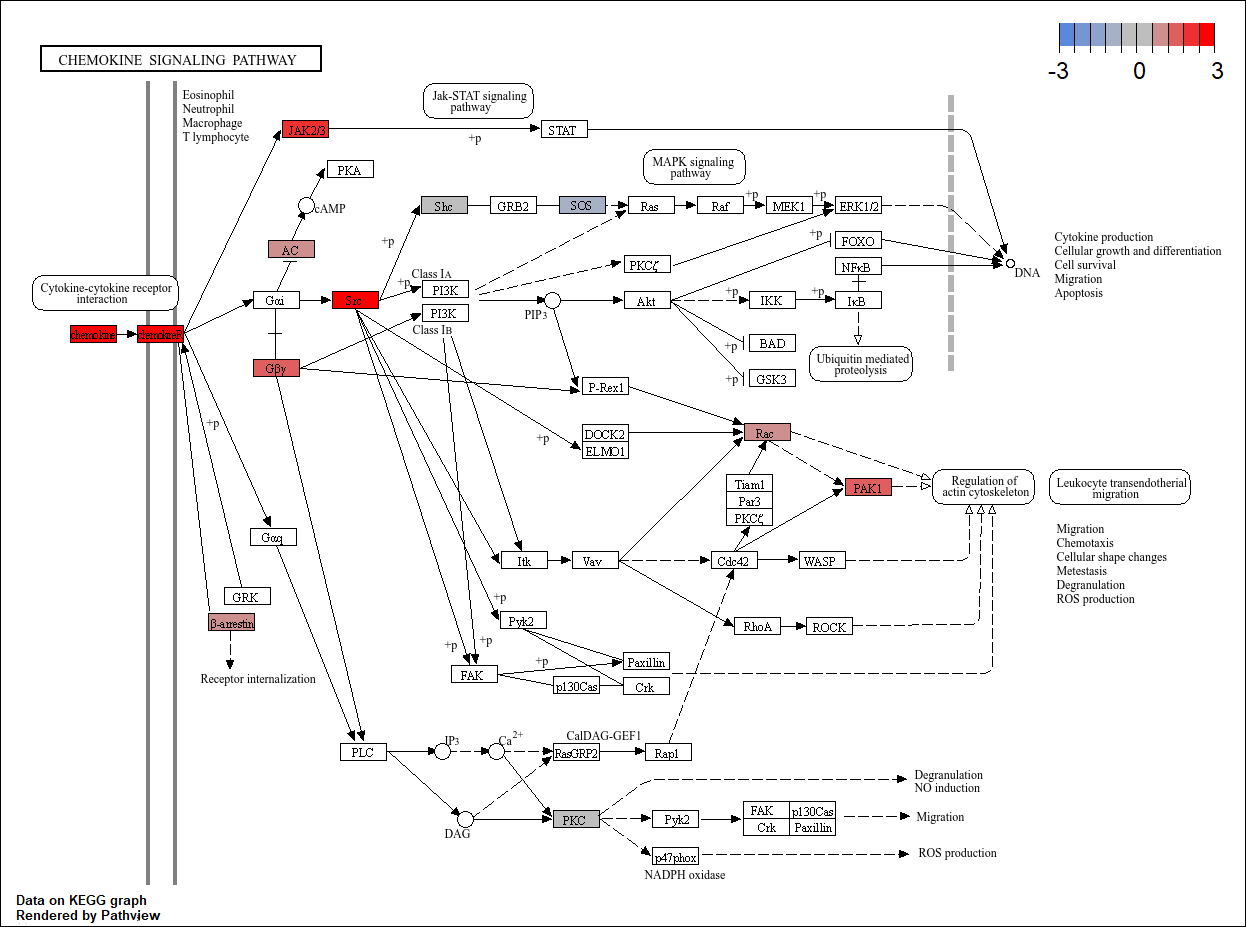


#### rno04210 “Apoptosis”


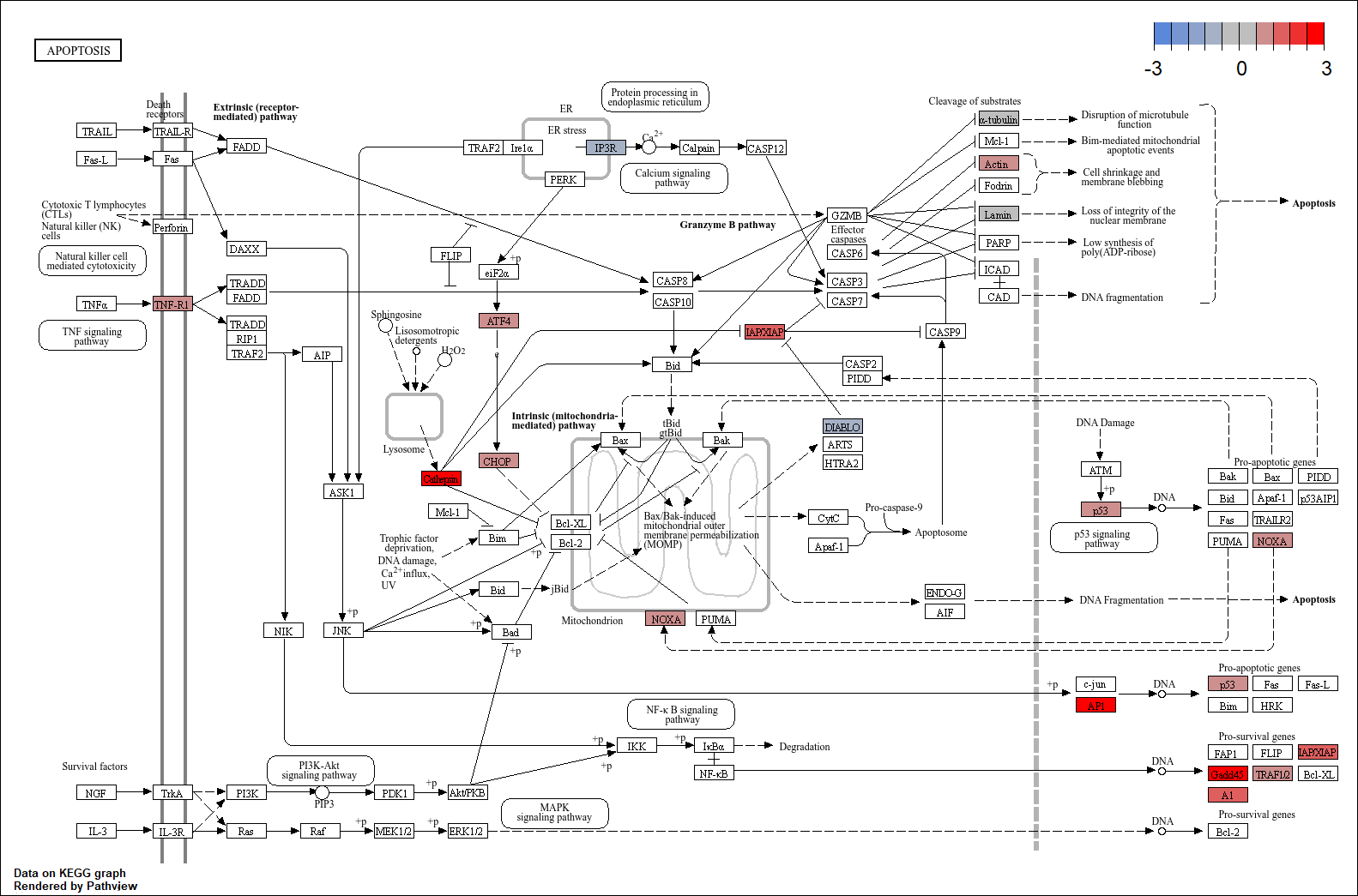


#### rno04514 “Cell adhesion molecules”


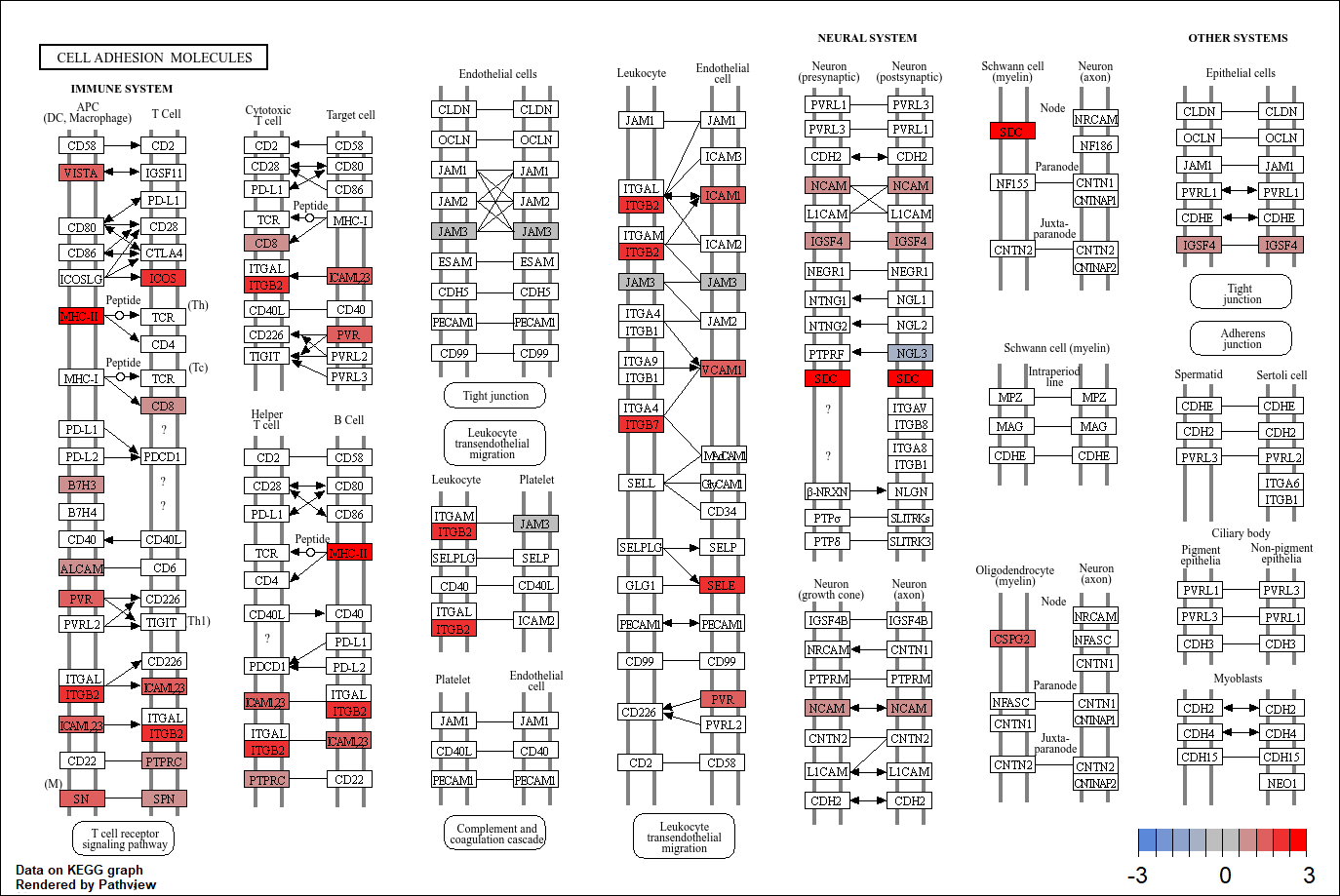


#### rno04650 “Natural killer cell mediated cytotoxicity


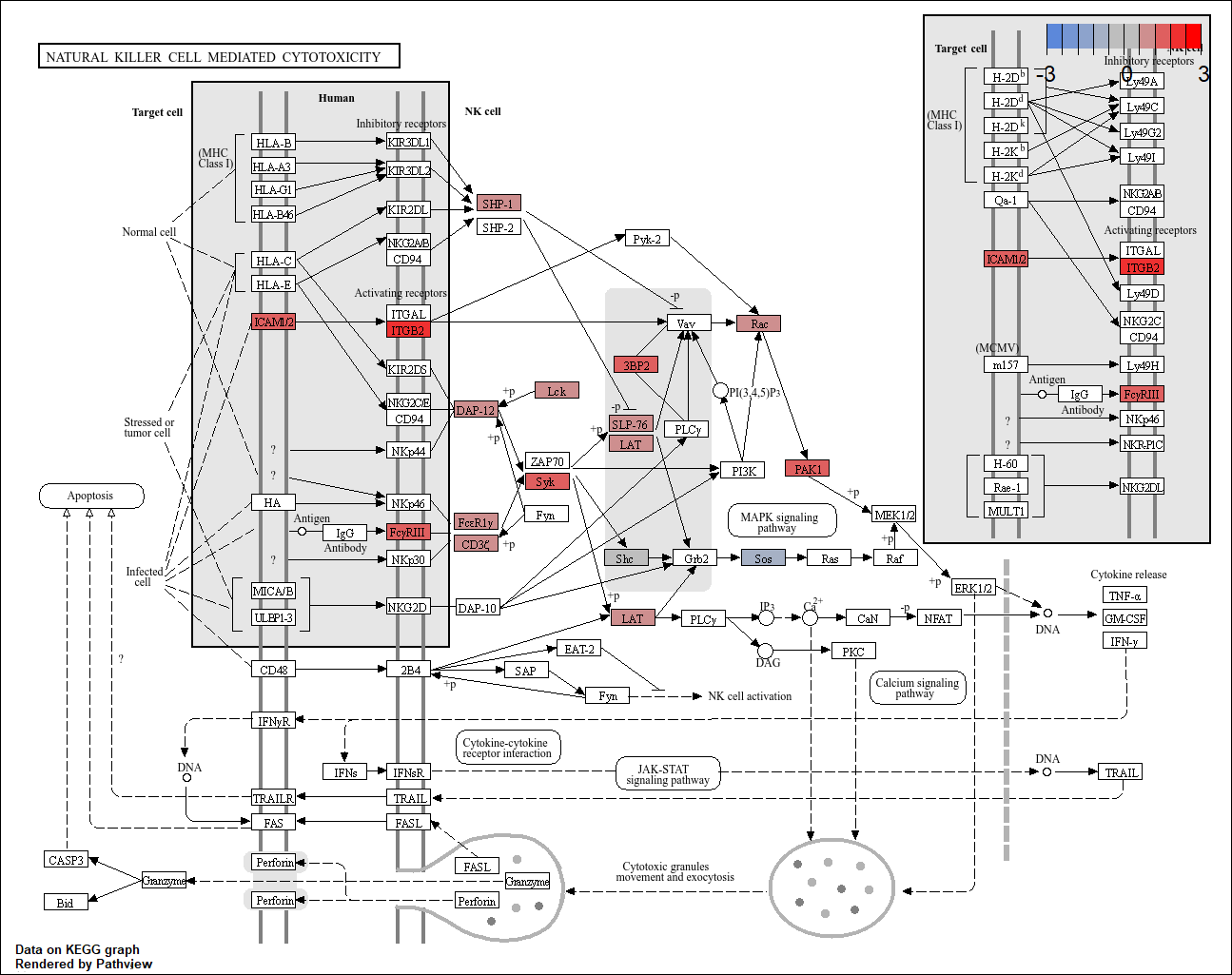


#### rno04657 “IL-17 signalling pathway


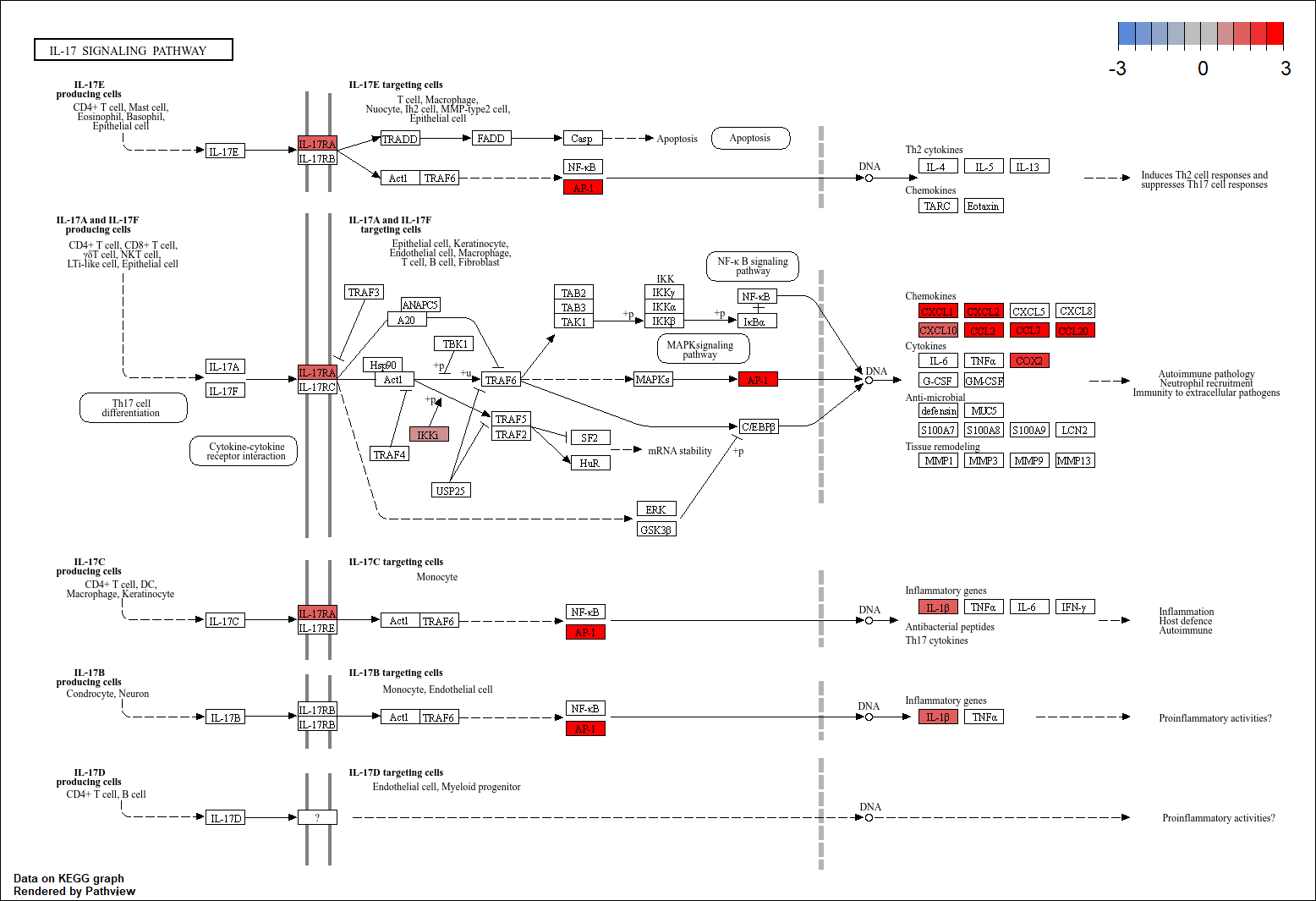


#### rno04659 “Th17 cell differentiation”


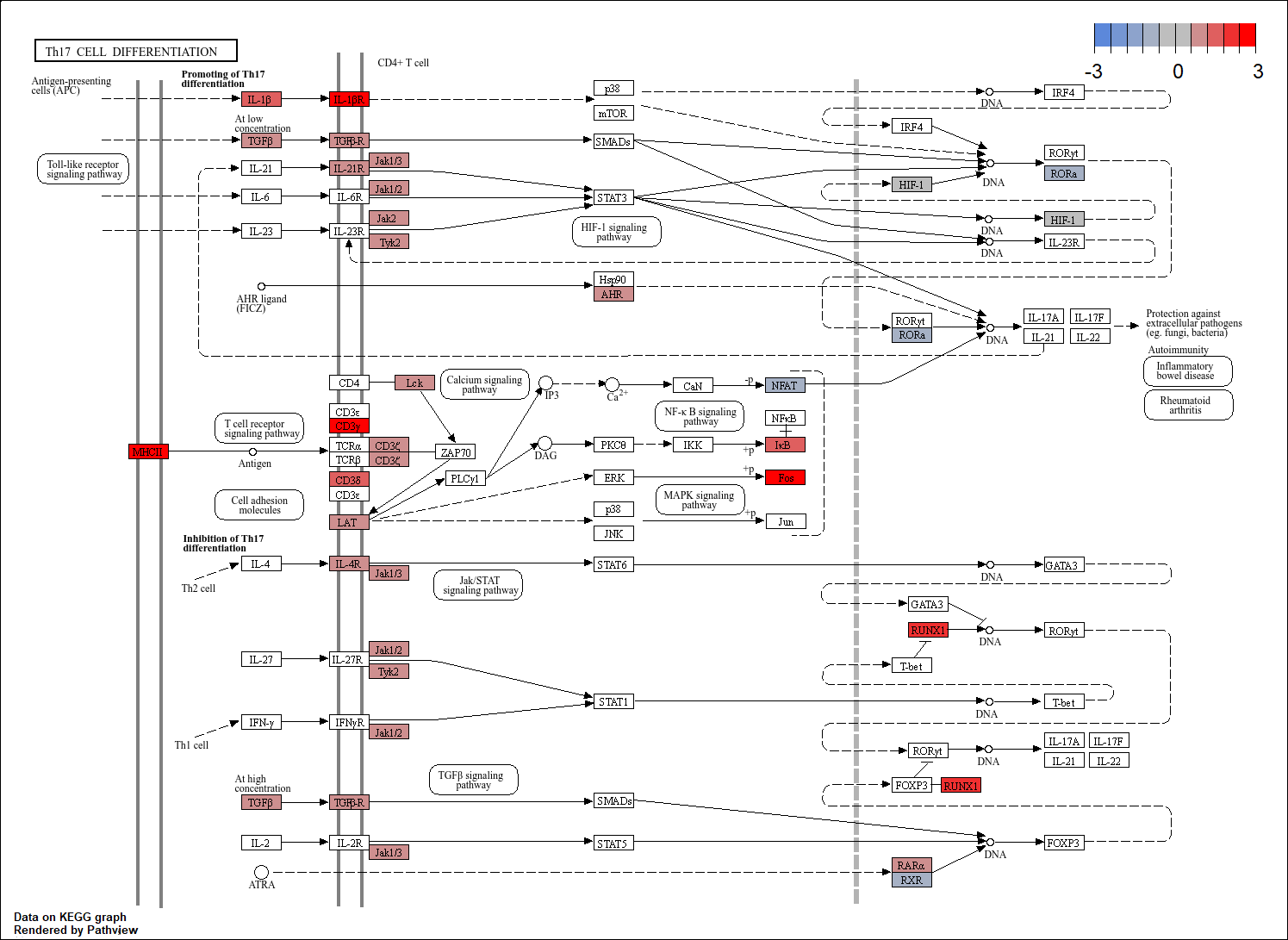


#### rno04662 “B cell receptor signalling pathway”


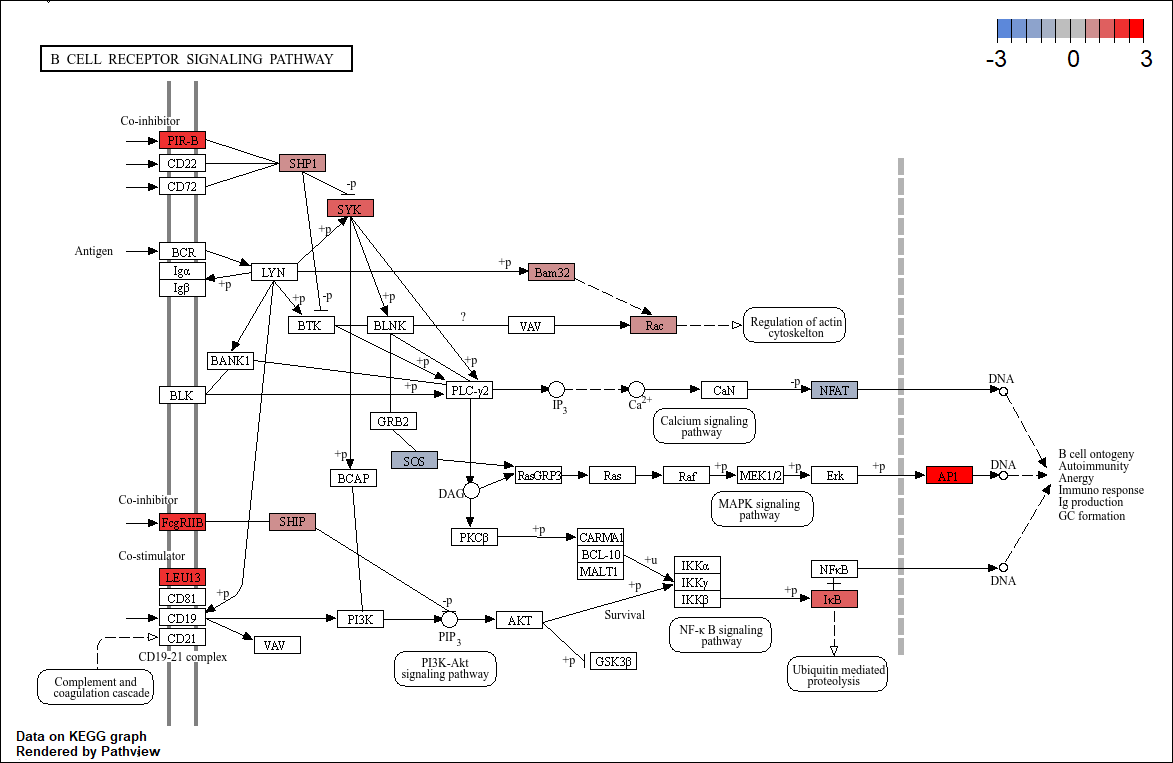


#### rno04666 “FcγR-mediated phagocytosis”


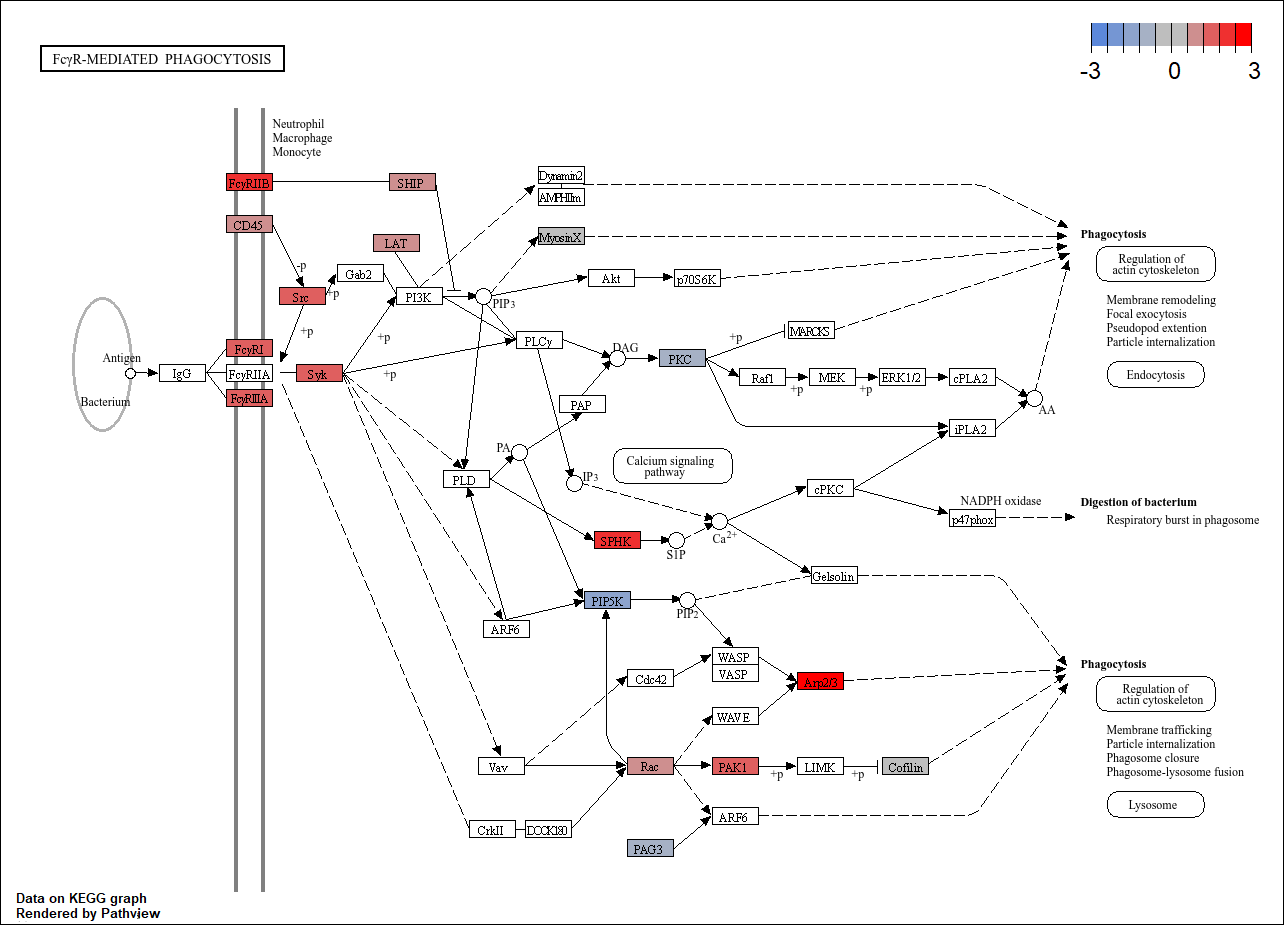


#### rno04668 “TNF signalling pathway”


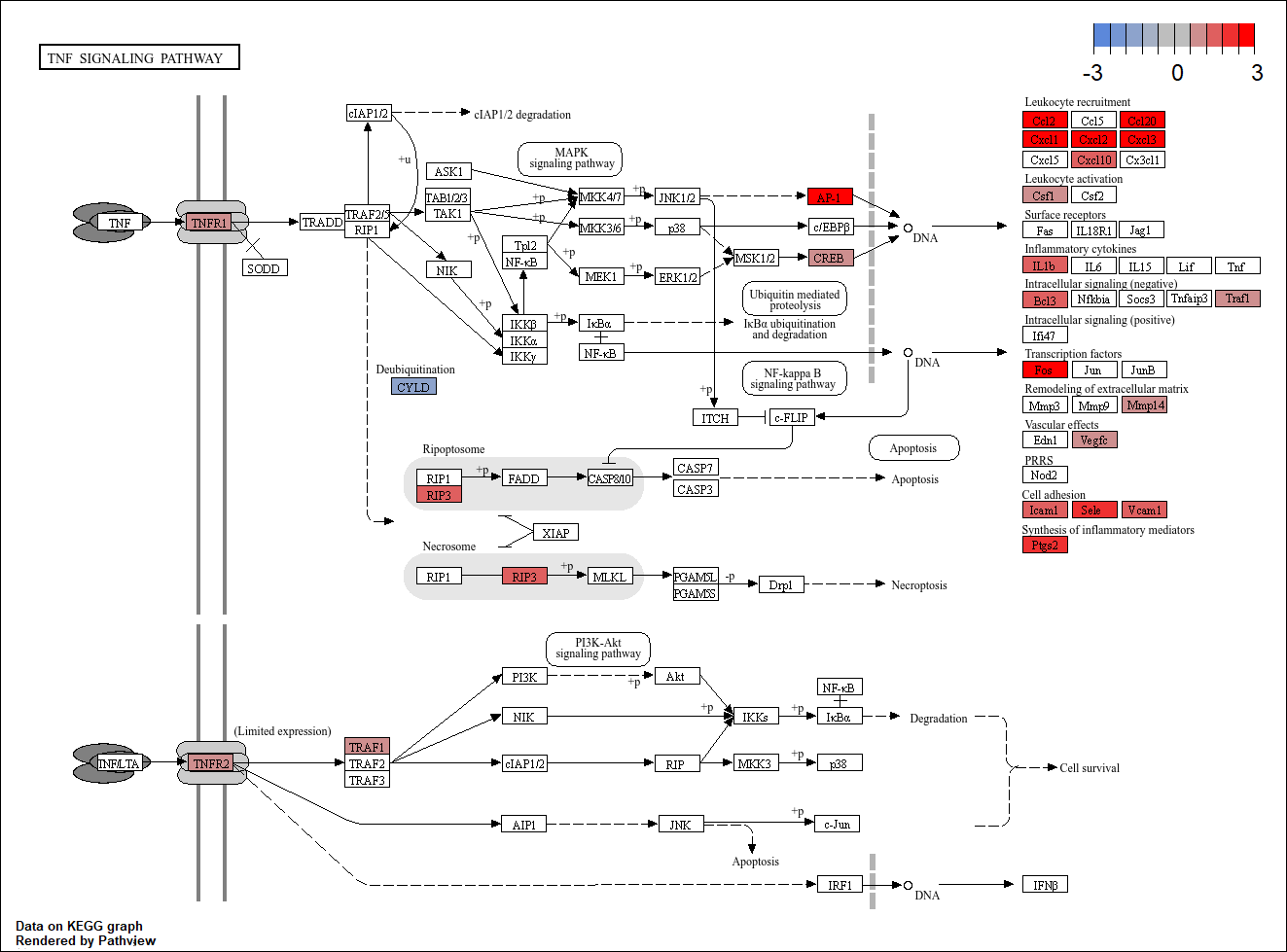


#### rno04670 “Leukocyte transendothelial migration”


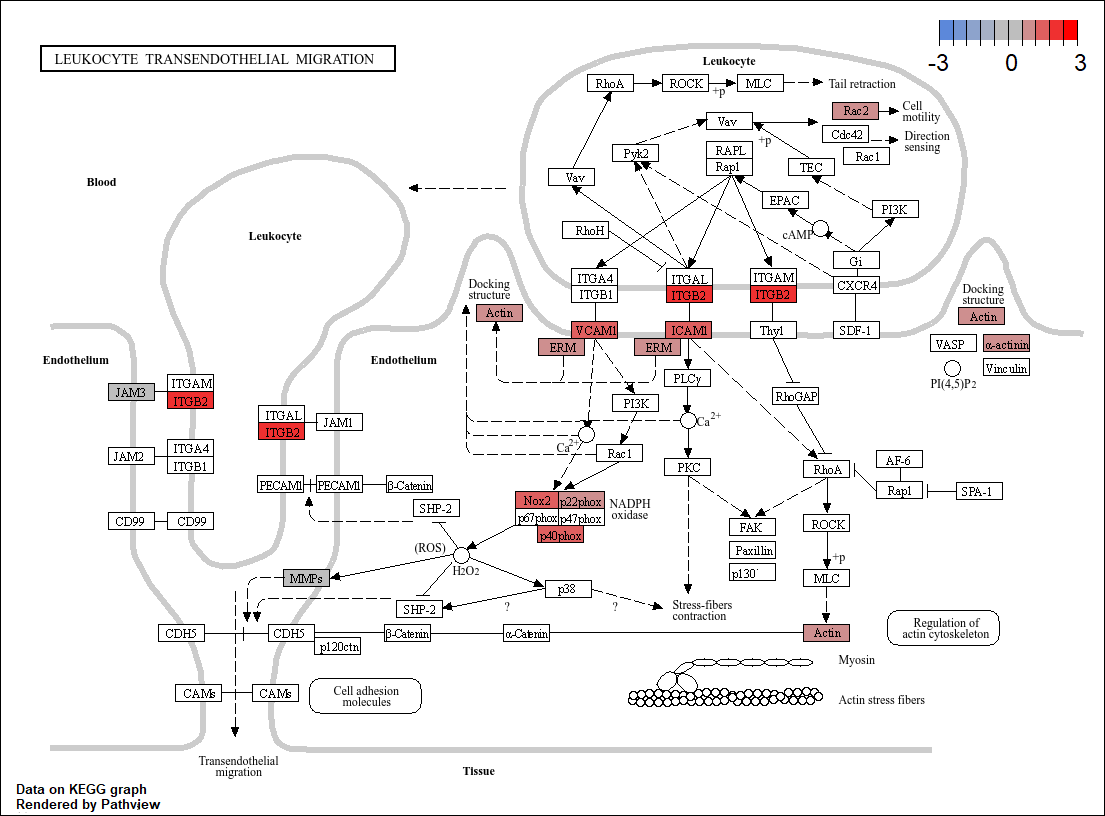


### Energy Metabolism

#### rno00010 “Glycolysis / gluconeogenesis”


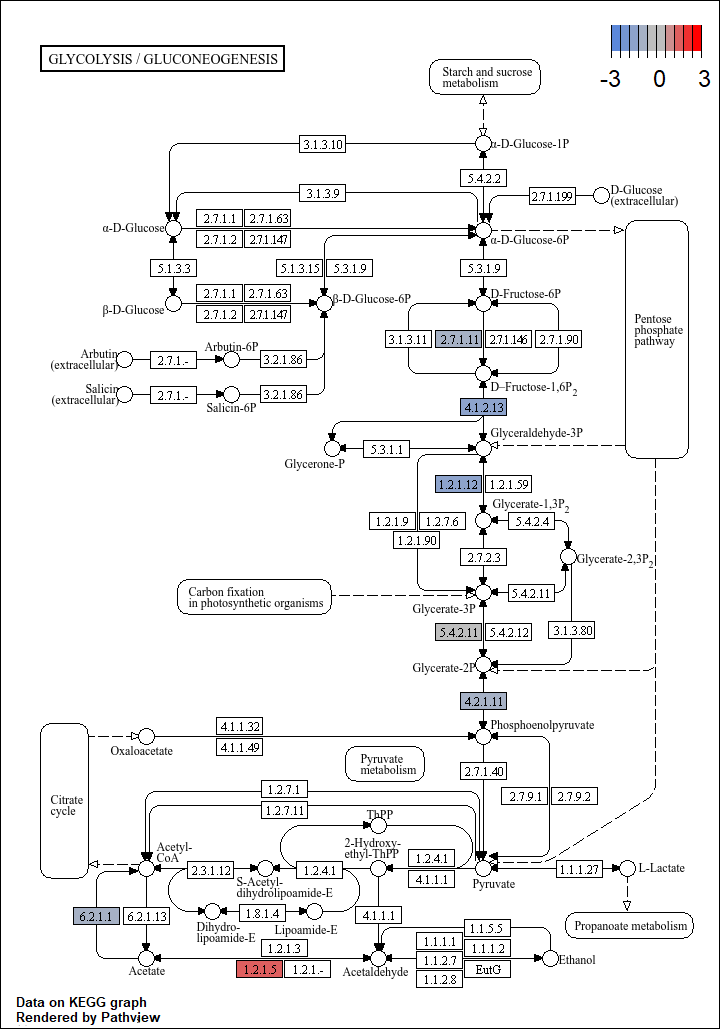


#### rno00071”Fatty acid degradation”


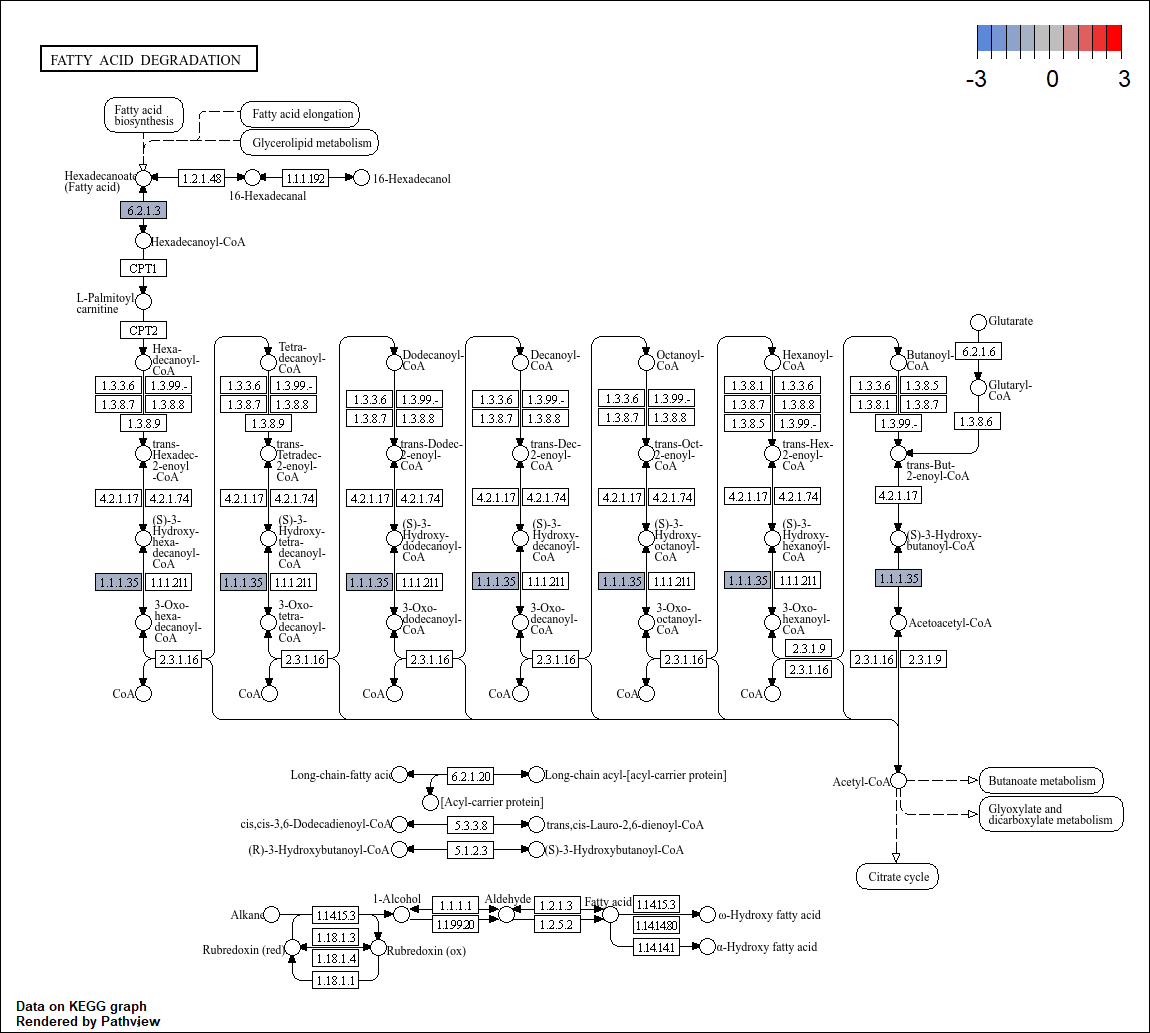


#### rno00190 “Oxidative phosphorylation”


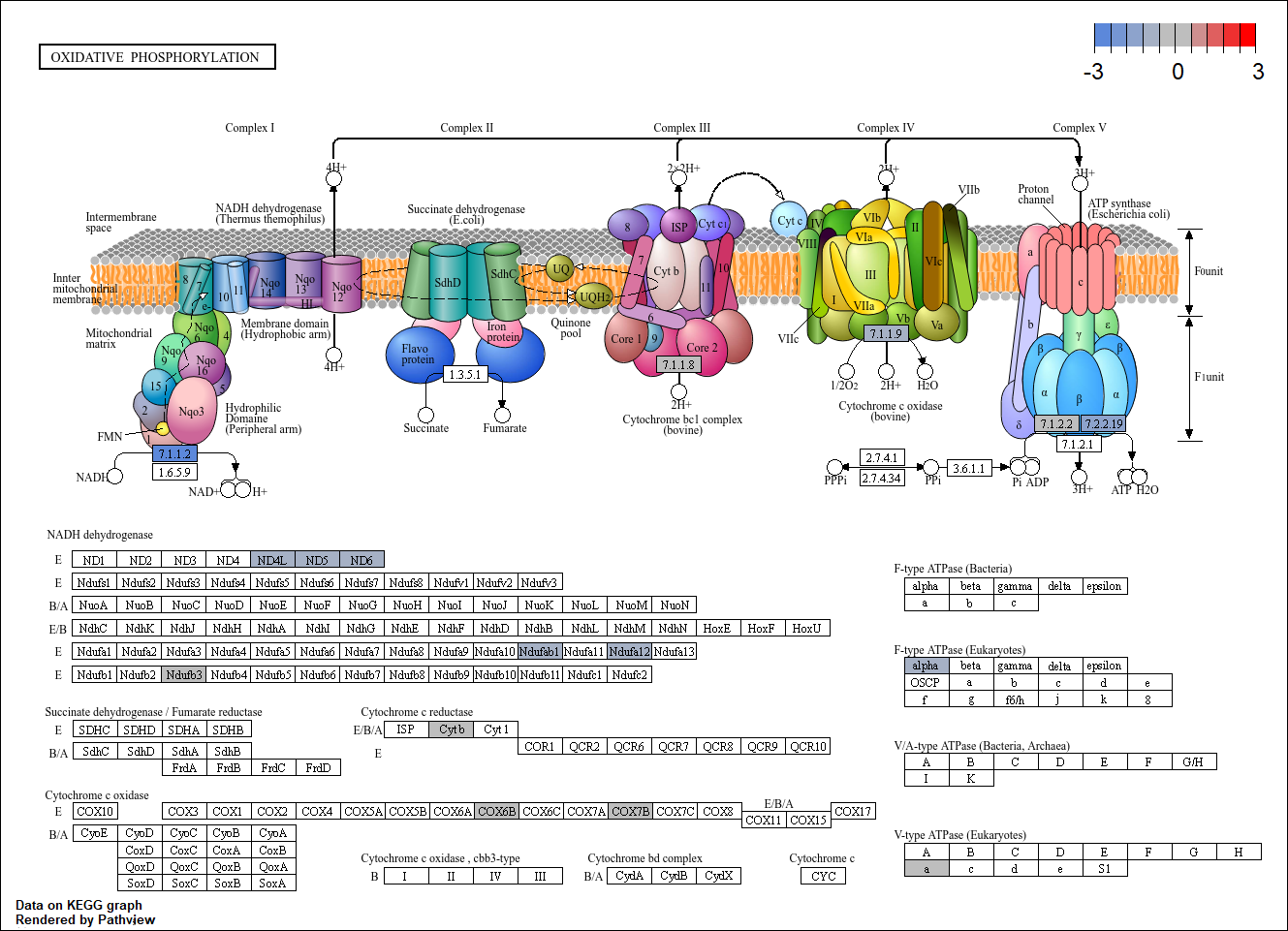


#### rno00640 “Propanoate metabolism”


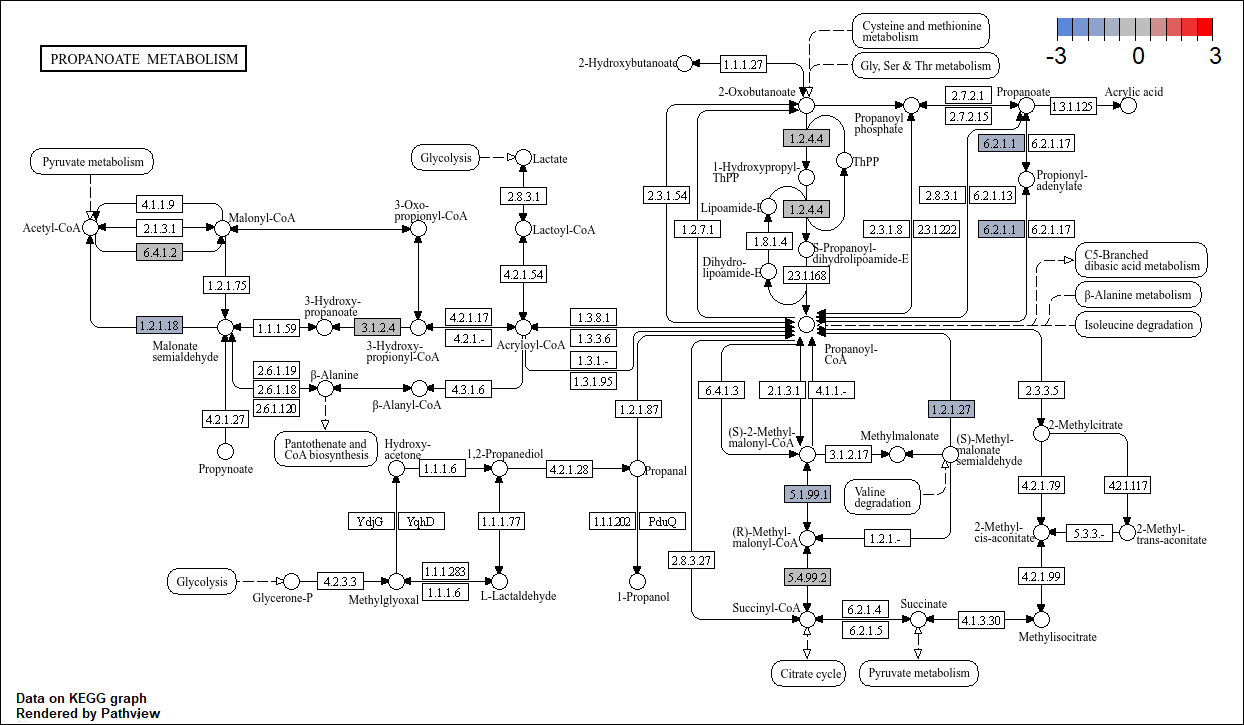


### Cardiac Function

#### rno04260 “Cardiac muscle contraction”


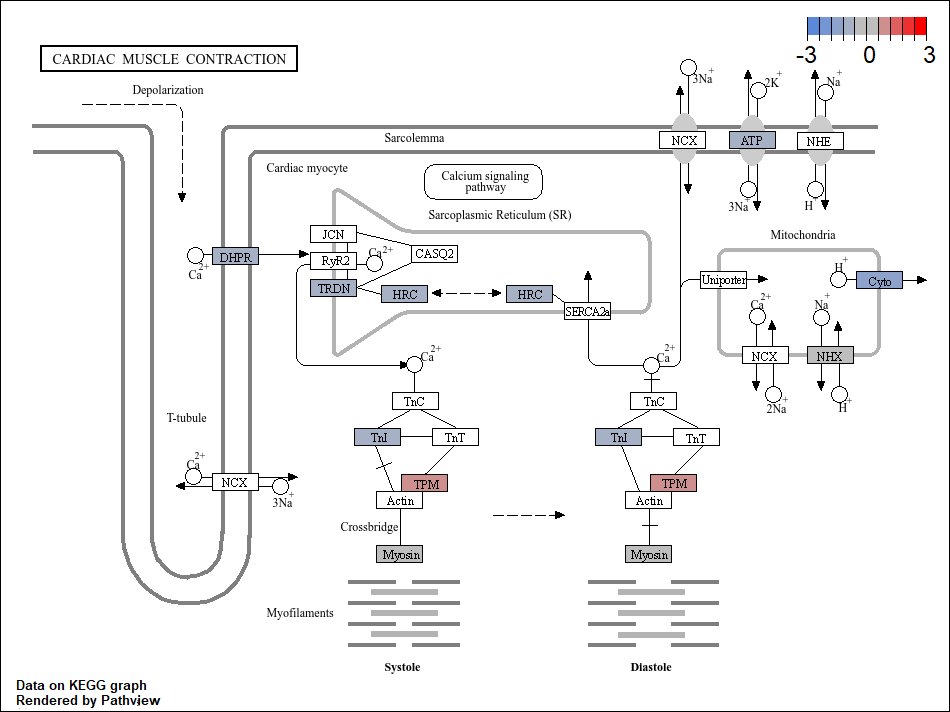

Supplement: Supplementary file 1 — Supplementary file1 (DOCX 1848 KB) [file 204_2024_3856_MOESM1_ESM.docx]
