# Supplementary material for: An integrated multi-omics analysis of the effects of the food processing-induced contaminant 2-monochloropropane-1,3-diol (2-MCPD) in rat heart
Source: Arch Toxicol. 2024 Sep 24;98(12):4033–45. doi: 10.1007/s00204-024-03856-6 (PMC11496350; doi:10.1007/s00204-024-03856-6)
Supplement: Supplementary file 2 — Supplementary file2 (DOCX 109 KB) [file 204_2024_3856_MOESM2_ESM.docx]

# Supplemental Table 1. Body weight, heart weight, food consumption, and water consumption of rats in the 90-day study of oral 2-MCPD exposure.

|  | Control | 40 mg/kg  2-MCPD |
| --- | --- | --- |
| Final Body Weight (g) | 328.0 ± 5.10 | 312.9 ± 4.20 |
| Heart Wet Weight (g/100g BW) | 0.2709 ± 0.0041 | 0.3018 ± 0.0036* |
| Food Consumption (g/kg BW)^†^ | 51.59 ± 0.24 | 51.22 ± 0.52 |
| Water Intake (g/kg BW)^†^ | 56.21 ± 1.81 | 57.82 ± 1.00 |
| Estimated 2-MCPD Consumption (mg/kg BW)^†^ | 0.00 ± 0.00 | 34.15 ± 0.35 |

**^†^** Mean weights taken over the entire duration of the study.

* Denotes significantly different (*P* < 0.05) from controls.

# Supplemental Table 2. Incidence of non-neoplastic lesions in rats from the 90-day study of oral 2-MCPD exposure.

|  | Control | 40mg/kg  2-MCPD |
| --- | --- | --- |
| Left Atrium  Interstitial Vacuolation  Necrosis  Interstitial Cells  Cardiomyocyte Vacuolation  Inflammation  Necrosis w Inflammatory Cell Infiltrate  Fibrosis | 5/0/0/0/0  5/0/0/0/0  5/0/0/0/0  5/0/0/0/0  5/0/0/0/0  5/0/0/0/0  5/0/0/0/0 | 5/0/0/0/0  5/0/0/0/0  4/1/0/0/0  5/0/0/0/0  3/2/0/0/0  3/2/0/0/0  5/0/0/0/0 |
| Right Atrium  Interstitial Vacuolation  Necrosis  Interstitial Cells  Cardiomyocyte Vacuolation  Inflammation  Necrosis w Inflammatory Cell Infiltrate  Fibrosis | 5/0/0/0/0  5/0/0/0/0  5/0/0/0/0  5/0/0/0/0  5/0/0/0/0  5/0/0/0/0  5/0/0/0/0 | 4/0/0/0/0  4/0/0/0/0  4/0/0/0/0  4/0/0/0/0  4/0/0/0/0  4/0/0/0/0  4/0/0/0/0 |
| Left Ventricle  Interstitial Vacuolation  Necrosis  Interstitial Cells  Cardiomyocyte Vacuolation  Inflammation  Necrosis w Inflammatory Cell Infiltrate  Fibrosis | 6/0/0/0/0  5/1/0/0/0  6/0/0/0/0  6/0/0/0/0  6/0/0/0/0  4/2/0/0/0  6/0/0/0/0 | 0/0/1/5/0*  1/4/1/0/0*  0/0/3/2/1*  0/0/5/1/0*  4/1/1/0/0  0/0/1/3/2*  0/0/6/0/0* |
| Right Ventricle  Interstitial Vacuolation  Necrosis  Interstitial Cells  Cardiomyocyte Vacuolation  Inflammation  Necrosis w Inflammatory Cell Infiltrate  Fibrosis | 6/0/0/0/0  6/0/0/0/0  6/0/0/0/0  6/0/0/0/0  4/2/0/0/0  5/1/0/0/0  6/0/0/0/0 | 2/1/3/0/0*  5/1/0/0/0  0/2/2/2/0*  1/0/5/0/0*  3/3/0/0/0  0/1/2/1/1*  0/2/4/0/0* |
| Septum  Interstitial Vacuolation  Necrosis  Interstitial Cells  Cardiomyocyte Vacuolation  Inflammation  Necrosis w Inflammatory Cell Infiltrate  Fibrosis | 6/0/0/0/0  5/1/0/0/0  6/0/0/0/0  6/0/0/0/0  6/0/0/0/0  3/3/0/0/0  5/1/0/0/0 | 0/0/2/3/0*  0/2/3/0/0  0/0/3/2/0*  0/0/4/1/0*  1/4/0/0/0  0/0/3/2/0*  0/2/3/0/0* |

Counts are the number of rats observed with absent/mild/moderate/marked/severe lesions.

* Denoted those that are significantly different (*P* < 0.05) from controls.

# Supplemental Table 3. RNA-seq rat heart DEGs with 40 mg/kg BW/d 2-MCPD exposure.

| ENSEMBL | Gene | LFC | *P* value | FDR |
| --- | --- | --- | --- | --- |
| ENSRNOG00000000047 | Cd82 | 1.09 | <0.001 | 0.011 |
| ENSRNOG00000000239 | Ccl7 | 3.23 | <0.001 | <0.001 |
| ENSRNOG00000000451 | RT1-Ba | 1.04 | <0.001 | 0.002 |
| ENSRNOG00000000528 | Fgd2 | 1.52 | <0.001 | 0.005 |
| ENSRNOG00000000569 | Vsir | 1.43 | <0.001 | 0.002 |
| ENSRNOG00000000640 | Egr2 | 3.03 | <0.001 | 0.003 |
| ENSRNOG00000000827 | Ier3 | 1.14 | <0.001 | 0.004 |
| ENSRNOG00000000853 | Aif1 | 1.02 | <0.001 | 0.007 |
| ENSRNOG00000000855 | Lst1 | 1.72 | 0.001 | 0.024 |
| ENSRNOG00000000907 | Alox5ap | 1.10 | <0.001 | <0.001 |
| ENSRNOG00000000994 | Stxbp2 | 1.37 | 0.002 | 0.028 |
| ENSRNOG00000001005 | Fcer2 | -1.15 | <0.001 | 0.004 |
| ENSRNOG00000001224 | Itgb2 | 1.92 | 0.002 | 0.033 |
| ENSRNOG00000001304 | Bcr | 1.41 | 0.003 | 0.038 |
| ENSRNOG00000001414 | Serpine1 | 2.35 | <0.001 | <0.001 |
| ENSRNOG00000001427 | Orai2 | 1.15 | 0.001 | 0.027 |
| ENSRNOG00000001704 | Runx1 | 2.10 | <0.001 | 0.001 |
| ENSRNOG00000002052 | Ccdc80 | 1.25 | <0.001 | <0.001 |
| ENSRNOG00000002385 | Prg4 | 1.67 | <0.001 | <0.001 |
| ENSRNOG00000002418 | Tgfb2 | 1.57 | <0.001 | <0.001 |
| ENSRNOG00000002434 | Tmem100 | 1.90 | <0.001 | <0.001 |
| ENSRNOG00000002524 | Gpr37 | 2.79 | 0.002 | 0.035 |
| ENSRNOG00000002643 | Ugdh | 1.40 | 0.002 | 0.034 |
| ENSRNOG00000002653 | Kcnk2 | -1.13 | <0.001 | 0.002 |
| ENSRNOG00000002746 | Fstl1 | 1.14 | <0.001 | 0.005 |
| ENSRNOG00000002810 | Gfpt2 | 1.41 | <0.001 | 0.014 |
| ENSRNOG00000002926 | Uap1 | 1.48 | <0.001 | 0.007 |
| ENSRNOG00000003120 | Prelp | 1.01 | <0.001 | 0.018 |
| ENSRNOG00000003357 | Col3a1 | 1.61 | <0.001 | <0.001 |
| ENSRNOG00000003486 | Mnda | 1.39 | <0.001 | 0.008 |
| ENSRNOG00000003533 | Clcn4 | -1.60 | 0.002 | 0.034 |
| ENSRNOG00000003546 | Tnfrsf12a | 1.65 | 0.002 | 0.028 |
| ENSRNOG00000003622 | Cybb | 1.21 | <0.001 | 0.004 |
| ENSRNOG00000003703 | Mcm6 | 1.03 | <0.001 | 0.007 |
| ENSRNOG00000003715 | Srpx2 | 1.81 | <0.001 | 0.019 |
| ENSRNOG00000003745 | Atf3 | 2.22 | <0.001 | 0.008 |
| ENSRNOG00000003778 | Opn3 | 2.31 | 0.001 | 0.023 |
| ENSRNOG00000003802 | Pttg1 | 1.16 | 0.003 | 0.045 |
| ENSRNOG00000003897 | Col1a1 | 1.55 | <0.001 | <0.001 |
| ENSRNOG00000003984 | Apln | -1.10 | <0.001 | 0.002 |
| ENSRNOG00000004078 | Eno3 | -1.16 | <0.001 | 0.002 |
| ENSRNOG00000004147 | Abca8a | -1.01 | <0.001 | 0.010 |
| ENSRNOG00000004229 | Tac3 | 4.90 | <0.001 | <0.001 |
| ENSRNOG00000004273 | Ifitm1 | 1.86 | <0.001 | <0.001 |
| ENSRNOG00000004578 | Cthrc1 | 4.15 | <0.001 | 0.004 |
| ENSRNOG00000004589 | Galnt16 | 1.12 | <0.001 | 0.008 |
| ENSRNOG00000004624 | Rnd3 | 1.09 | <0.001 | 0.004 |
| ENSRNOG00000004649 | Il1b | 1.70 | 0.002 | 0.032 |
| ENSRNOG00000004699 | Fibin | 1.19 | <0.001 | 0.002 |
| ENSRNOG00000005214 | Plek | 1.53 | <0.001 | 0.003 |
| ENSRNOG00000005243 | Pop1 | 1.28 | 0.003 | 0.046 |
| ENSRNOG00000005248 | Slc1a4 | 1.36 | <0.001 | 0.012 |
| ENSRNOG00000005378 | Gna15 | 1.54 | 0.003 | 0.045 |
| ENSRNOG00000005387 | Rbm3 | 1.01 | <0.001 | 0.001 |
| ENSRNOG00000005695 | Mgp | 1.12 | <0.001 | 0.005 |
| ENSRNOG00000005825 | Lyz2 | 1.94 | <0.001 | <0.001 |
| ENSRNOG00000005854 | Angpt1 | -1.46 | <0.001 | 0.003 |
| ENSRNOG00000005871 | Il1rn | 2.60 | <0.001 | 0.002 |
| ENSRNOG00000005935 | A3galt2 | 1.81 | <0.001 | 0.004 |
| ENSRNOG00000005965 | Irak4 | 1.10 | 0.002 | 0.034 |
| ENSRNOG00000006094 | Cd44 | 1.12 | 0.003 | 0.041 |
| ENSRNOG00000006151 | Reg3b | 5.07 | <0.001 | <0.001 |
| ENSRNOG00000006231 | Ptpro | 1.20 | <0.001 | 0.003 |
| ENSRNOG00000006460 | Amdhd2 | 1.30 | 0.003 | 0.040 |
| ENSRNOG00000006553 | Bnc2 | 2.05 | <0.001 | <0.001 |
| ENSRNOG00000007159 | Ccl2 | 3.01 | <0.001 | 0.020 |
| ENSRNOG00000007290 | Atp1a2 | -1.08 | <0.001 | 0.002 |
| ENSRNOG00000007302 | Fbn1 | 1.53 | <0.001 | <0.001 |
| ENSRNOG00000007319 | Trib3 | 2.93 | <0.001 | <0.001 |
| ENSRNOG00000007437 | Irf5 | 1.50 | <0.001 | 0.007 |
| ENSRNOG00000007546 | Asns | 2.92 | 0.002 | 0.028 |
| ENSRNOG00000007613 | C1qtnf5 | 1.11 | 0.003 | 0.038 |
| ENSRNOG00000007679 | Cyth4 | 1.23 | <0.001 | 0.003 |
| ENSRNOG00000008015 | Fos | 2.75 | <0.001 | <0.001 |
| ENSRNOG00000008045 | Slamf9 | 1.80 | <0.001 | 0.003 |
| ENSRNOG00000008115 | Arhgap11a | 1.16 | 0.002 | 0.037 |
| ENSRNOG00000008141 | Nppb | 1.35 | <0.001 | 0.005 |
| ENSRNOG00000008187 | Ubash3b | 1.46 | 0.003 | 0.041 |
| ENSRNOG00000008336 | Tnfrsf11b | 3.27 | 0.001 | 0.026 |
| ENSRNOG00000008409 | Myo1f | 1.16 | <0.001 | 0.004 |
| ENSRNOG00000008439 | Gprc5d | 1.86 | 0.001 | 0.022 |
| ENSRNOG00000008680 | Loxl1 | 1.69 | <0.001 | <0.001 |
| ENSRNOG00000008736 | Slamf8 | 1.17 | <0.001 | 0.010 |
| ENSRNOG00000008816 | Gpnmb | 2.56 | <0.001 | <0.001 |
| ENSRNOG00000008837 | Ass1 | 2.04 | 0.001 | 0.022 |
| ENSRNOG00000009197 | Asb4 | -1.26 | 0.002 | 0.031 |
| ENSRNOG00000009211 | C3ar1 | 2.76 | 0.003 | 0.038 |
| ENSRNOG00000009331 | Hck | 1.60 | <0.001 | 0.004 |
| ENSRNOG00000009334 | Knstrn | 1.54 | 0.004 | 0.047 |
| ENSRNOG00000009341 | Hivep3 | 1.41 | <0.001 | 0.004 |
| ENSRNOG00000009594 | Snai1 | 1.20 | 0.002 | 0.033 |
| ENSRNOG00000009724 | Tstd2 | -1.20 | 0.002 | 0.033 |
| ENSRNOG00000009730 | Cyp7b1 | 2.69 | <0.001 | 0.020 |
| ENSRNOG00000009848 | Il18 | 1.33 | <0.001 | 0.015 |
| ENSRNOG00000009946 | Ldlr | 1.33 | <0.001 | 0.008 |
| ENSRNOG00000010047 | Ddit4l | -1.36 | <0.001 | 0.008 |
| ENSRNOG00000010092 | Magix | -1.06 | <0.001 | 0.019 |
| ENSRNOG00000010208 | Timp1 | 3.68 | <0.001 | <0.001 |
| ENSRNOG00000010319 | Lcp1 | 1.22 | <0.001 | 0.005 |
| ENSRNOG00000010362 | Anxa2 | 1.03 | <0.001 | <0.001 |
| ENSRNOG00000010466 | Chpf2 | 1.24 | 0.001 | 0.022 |
| ENSRNOG00000010626 | Sphk1 | 2.26 | <0.001 | 0.012 |
| ENSRNOG00000010645 | Lgals3 | 1.88 | <0.001 | <0.001 |
| ENSRNOG00000010833 | Mthfd2 | 1.88 | <0.001 | <0.001 |
| ENSRNOG00000010986 | - | 1.02 | <0.001 | 0.008 |
| ENSRNOG00000010994 | Has1 | 2.99 | <0.001 | 0.004 |
| ENSRNOG00000011296 | Cenpn | 1.26 | 0.003 | 0.044 |
| ENSRNOG00000011406 | Ccl4 | 1.13 | 0.002 | 0.032 |
| ENSRNOG00000011631 | Fst | 2.66 | <0.001 | 0.004 |
| ENSRNOG00000011644 | Slc1a7 | 2.40 | <0.001 | 0.010 |
| ENSRNOG00000011647 | S100a6 | 1.02 | <0.001 | <0.001 |
| ENSRNOG00000011821 | S100a4 | 1.39 | <0.001 | <0.001 |
| ENSRNOG00000012094 | Ltbp2 | 3.03 | <0.001 | <0.001 |
| ENSRNOG00000012098 | Adcyap1r1 | 1.58 | <0.001 | <0.001 |
| ENSRNOG00000012160 | Syk | 1.24 | 0.001 | 0.023 |
| ENSRNOG00000012172 | Spi1 | 1.55 | <0.001 | 0.005 |
| ENSRNOG00000012208 | Itgb7 | 2.21 | <0.001 | 0.019 |
| ENSRNOG00000012390 | Npw | 3.56 | <0.001 | 0.007 |
| ENSRNOG00000012458 | Cyp2e1 | -1.77 | <0.001 | 0.002 |
| ENSRNOG00000012471 | Thbs4 | 3.84 | <0.001 | <0.001 |
| ENSRNOG00000012494 | Kctd14 | 1.30 | 0.003 | 0.046 |
| ENSRNOG00000012616 | Ppt1 | 1.06 | <0.001 | 0.002 |
| ENSRNOG00000012660 | Postn | 3.45 | <0.001 | <0.001 |
| ENSRNOG00000012749 | C1qb | 1.03 | <0.001 | 0.006 |
| ENSRNOG00000012807 | C1qa | 1.23 | <0.001 | 0.014 |
| ENSRNOG00000012881 | Fgl2 | 1.60 | <0.001 | 0.002 |
| ENSRNOG00000013014 | Cyba | 1.10 | <0.001 | 0.015 |
| ENSRNOG00000013024 | Csgalnact1 | 1.03 | <0.001 | 0.008 |
| ENSRNOG00000013231 | Ptafr | 1.14 | <0.001 | 0.016 |
| ENSRNOG00000013306 | Pcdh20 | -2.13 | <0.001 | 0.017 |
| ENSRNOG00000013330 | Cdhr1 | 2.64 | 0.003 | 0.038 |
| ENSRNOG00000013564 | Dok3 | 1.76 | 0.002 | 0.037 |
| ENSRNOG00000013653 | Pdlim7 | 1.06 | 0.002 | 0.029 |
| ENSRNOG00000013744 | Akip1 | 1.22 | <0.001 | <0.001 |
| ENSRNOG00000013747 | Sh3bp2 | 1.76 | <0.001 | 0.006 |
| ENSRNOG00000013794 | Rbp1 | 1.29 | <0.001 | 0.003 |
| ENSRNOG00000013862 | Dusp2 | 1.67 | <0.001 | 0.008 |
| ENSRNOG00000013914 | Gpr132 | 3.03 | <0.001 | 0.014 |
| ENSRNOG00000013917 | Igsf10 | 1.43 | <0.001 | 0.002 |
| ENSRNOG00000013981 | Ptpn5 | -2.51 | 0.002 | 0.029 |
| ENSRNOG00000013987 | Sbno2 | 1.58 | 0.001 | 0.022 |
| ENSRNOG00000014117 | Hmox1 | 2.16 | <0.001 | <0.001 |
| ENSRNOG00000014202 | Snx20 | 1.71 | 0.001 | 0.025 |
| ENSRNOG00000014258 | Rab32 | 1.15 | <0.001 | 0.011 |
| ENSRNOG00000014288 | Fn1 | 1.39 | <0.001 | <0.001 |
| ENSRNOG00000014320 | Inhba | 1.67 | 0.002 | 0.035 |
| ENSRNOG00000014333 | Vcam1 | 1.43 | <0.001 | 0.003 |
| ENSRNOG00000014387 | Chac1 | 5.43 | <0.001 | <0.001 |
| ENSRNOG00000014426 | Lox | 1.71 | <0.001 | <0.001 |
| ENSRNOG00000014504 | Il1r1 | 2.75 | 0.002 | 0.030 |
| ENSRNOG00000014532 | Lbp | 1.55 | <0.001 | 0.001 |
| ENSRNOG00000014653 | Arl11 | 1.33 | <0.001 | 0.019 |
| ENSRNOG00000014816 | Slc1a1 | 1.08 | <0.001 | 0.007 |
| ENSRNOG00000014956 | Slc11a1 | 1.36 | 0.003 | 0.042 |
| ENSRNOG00000015159 | Slc9a3 | 2.95 | <0.001 | 0.008 |
| ENSRNOG00000015505 | Mfap5 | 1.75 | <0.001 | <0.001 |
| ENSRNOG00000015514 | Bcat1 | 2.93 | <0.001 | <0.001 |
| ENSRNOG00000015904 | Wfdc1 | -1.14 | <0.001 | <0.001 |
| ENSRNOG00000015906 | Tgif1 | 1.98 | <0.001 | 0.005 |
| ENSRNOG00000015941 | Fkbp10 | 1.00 | 0.002 | 0.032 |
| ENSRNOG00000015992 | Ccl20 | 3.53 | <0.001 | 0.017 |
| ENSRNOG00000016085 | Mpzl2 | 2.18 | 0.002 | 0.034 |
| ENSRNOG00000016151 | Ankrd23 | 1.52 | <0.001 | 0.002 |
| ENSRNOG00000016257 | Cotl1 | 1.60 | <0.001 | <0.001 |
| ENSRNOG00000016483 | Myo16 | -1.76 | <0.001 | 0.020 |
| ENSRNOG00000016581 | Serpinb1a | 1.68 | <0.001 | 0.001 |
| ENSRNOG00000016643 | Lpcat2 | 1.29 | <0.001 | 0.015 |
| ENSRNOG00000016756 | Ptgir | 1.28 | 0.004 | 0.047 |
| ENSRNOG00000016758 | Loxl2 | 1.29 | <0.001 | 0.002 |
| ENSRNOG00000016825 | Polr1g | 1.06 | <0.001 | 0.011 |
| ENSRNOG00000016980 | Qprt | 2.54 | <0.001 | 0.014 |
| ENSRNOG00000017020 | Inpp5d | 1.13 | <0.001 | 0.004 |
| ENSRNOG00000017120 | Abhd2 | 1.73 | 0.003 | 0.038 |
| ENSRNOG00000017259 | Tacc3 | 1.22 | 0.002 | 0.032 |
| ENSRNOG00000017445 | Tubb2b | 1.18 | <0.001 | 0.016 |
| ENSRNOG00000017477 | Mmp23 | 1.07 | <0.001 | 0.018 |
| ENSRNOG00000017786 | Acta1 | 1.05 | <0.001 | 0.006 |
| ENSRNOG00000017869 | Irf8 | 1.23 | <0.001 | 0.004 |
| ENSRNOG00000017874 | Cd53 | 1.09 | 0.001 | 0.025 |
| ENSRNOG00000018092 | Cd83 | 1.13 | 0.001 | 0.024 |
| ENSRNOG00000018251 | Mrc1 | 1.35 | <0.001 | 0.001 |
| ENSRNOG00000018285 | Kcna2 | -1.13 | 0.002 | 0.028 |
| ENSRNOG00000018371 | Tubb6 | 1.89 | <0.001 | <0.001 |
| ENSRNOG00000018414 | Csf1r | 1.19 | <0.001 | 0.018 |
| ENSRNOG00000018484 | Plk3 | 1.38 | 0.001 | 0.022 |
| ENSRNOG00000018646 | Hbegf | 1.12 | 0.003 | 0.042 |
| ENSRNOG00000018669 | Jak3 | 1.01 | <0.001 | 0.011 |
| ENSRNOG00000018681 | Nes | 1.02 | 0.002 | 0.036 |
| ENSRNOG00000018690 | Rgs17 | -1.64 | 0.002 | 0.037 |
| ENSRNOG00000018715 | Clec10a | 1.35 | <0.001 | <0.001 |
| ENSRNOG00000018778 | Cadm1 | 1.15 | 0.003 | 0.042 |
| ENSRNOG00000019161 | Cpeb1 | 1.66 | <0.001 | 0.016 |
| ENSRNOG00000019211 | Olfml3 | 1.31 | <0.001 | <0.001 |
| ENSRNOG00000019270 | P2ry6 | 1.12 | 0.002 | 0.036 |
| ENSRNOG00000019365 | Ablim3 | -1.17 | <0.001 | 0.019 |
| ENSRNOG00000019387 | Ifi30 | 1.07 | <0.001 | 0.001 |
| ENSRNOG00000019422 | Egr1 | 2.12 | <0.001 | <0.001 |
| ENSRNOG00000019430 | Coro1a | 1.22 | <0.001 | <0.001 |
| ENSRNOG00000019440 | Kcnn4 | 1.65 | <0.001 | 0.004 |
| ENSRNOG00000019587 | Ptprn | 2.40 | <0.001 | 0.008 |
| ENSRNOG00000019648 | Col6a3 | 1.35 | <0.001 | 0.004 |
| ENSRNOG00000019680 | Mst1 | -1.67 | <0.001 | 0.008 |
| ENSRNOG00000019780 | Sypl2 | 1.82 | <0.001 | 0.018 |
| ENSRNOG00000019854 | Napsa | 1.55 | <0.001 | 0.011 |
| ENSRNOG00000019890 | Folr2 | 1.14 | <0.001 | 0.004 |
| ENSRNOG00000020030 | Crlf1 | 1.73 | <0.001 | <0.001 |
| ENSRNOG00000020251 | Art1 | -1.29 | <0.001 | 0.004 |
| ENSRNOG00000020300 | Lsp1 | 1.73 | <0.001 | <0.001 |
| ENSRNOG00000020465 | Ripk3 | 1.27 | 0.002 | 0.030 |
| ENSRNOG00000020587 | Efemp2 | 1.01 | <0.001 | 0.005 |
| ENSRNOG00000020679 | Icam1 | 1.56 | <0.001 | 0.002 |
| ENSRNOG00000020848 | Coq8b | 1.10 | <0.001 | 0.005 |
| ENSRNOG00000020867 | Numbl | 1.07 | <0.001 | 0.006 |
| ENSRNOG00000020991 | Ms4a6a | 1.36 | 0.002 | 0.029 |
| ENSRNOG00000021027 | Dbp | 1.64 | <0.001 | 0.004 |
| ENSRNOG00000021062 | Fxyd5 | 1.19 | <0.001 | 0.004 |
| ENSRNOG00000021084 | - | 2.06 | 0.001 | 0.026 |
| ENSRNOG00000021104 | Emp3 | 1.13 | <0.001 | 0.005 |
| ENSRNOG00000021155 | Ctsk | 1.20 | <0.001 | 0.002 |
| ENSRNOG00000021199 | Fcgr1a | 1.76 | <0.001 | 0.004 |
| ENSRNOG00000021243 | Siglec1 | 1.41 | 0.001 | 0.024 |
| ENSRNOG00000021445 | Tas2r120 | -1.77 | 0.002 | 0.028 |
| ENSRNOG00000021560 | Cass4 | 1.18 | 0.003 | 0.042 |
| ENSRNOG00000021663 | Vxn | 1.67 | <0.001 | 0.020 |
| ENSRNOG00000022012 | Tnfrsf18 | 3.13 | <0.001 | 0.016 |
| ENSRNOG00000022256 | Cxcl10 | 1.63 | <0.001 | 0.012 |
| ENSRNOG00000022268 | Pnpla3 | -1.19 | <0.001 | 0.007 |
| ENSRNOG00000022657 | - | 1.13 | <0.001 | 0.013 |
| ENSRNOG00000022975 | Nfam1 | 1.29 | 0.001 | 0.022 |
| ENSRNOG00000023209 | Slamf7 | 1.17 | 0.001 | 0.025 |
| ENSRNOG00000023628 | Tmem106a | 1.29 | <0.001 | 0.016 |
| ENSRNOG00000023799 | Pak1ip1 | 1.25 | <0.001 | 0.007 |
| ENSRNOG00000024899 | Cxcl13 | 1.31 | 0.003 | 0.046 |
| ENSRNOG00000025001 | Pcolce | 1.14 | <0.001 | 0.003 |
| ENSRNOG00000025130 | Ltk | 2.62 | 0.003 | 0.044 |
| ENSRNOG00000026306 | Clec5a | 3.16 | <0.001 | <0.001 |
| ENSRNOG00000026548 | Dhrs7c | -1.18 | <0.001 | 0.005 |
| ENSRNOG00000026604 | Cercam | 1.06 | 0.004 | 0.047 |
| ENSRNOG00000026647 | Cxcl16 | 1.04 | <0.001 | 0.001 |
| ENSRNOG00000027024 | Rgs16 | 1.31 | 0.002 | 0.029 |
| ENSRNOG00000027096 | Ctsw | 1.82 | 0.002 | 0.032 |
| ENSRNOG00000027811 | Lilrb4 | 2.00 | <0.001 | 0.019 |
| ENSRNOG00000027888 | Cmss1 | 1.35 | 0.003 | 0.042 |
| ENSRNOG00000028930 | Dab2 | 1.02 | <0.001 | <0.001 |
| ENSRNOG00000029145 | - | -1.24 | <0.001 | <0.001 |
| ENSRNOG00000029212 | Vcan | 1.63 | <0.001 | 0.004 |
| ENSRNOG00000029662 | Wdfy4 | 1.05 | 0.002 | 0.029 |
| ENSRNOG00000029682 | Clic1 | 1.55 | <0.001 | 0.008 |
| ENSRNOG00000029784 | Pak1 | 1.59 | <0.001 | 0.006 |
| ENSRNOG00000029911 | Cilp | 2.39 | <0.001 | 0.003 |
| ENSRNOG00000029980 | Zbtb16 | -1.87 | <0.001 | 0.005 |
| ENSRNOG00000030012 | Clec4a2 | 2.79 | 0.003 | 0.041 |
| ENSRNOG00000030330 | - | -1.49 | 0.002 | 0.030 |
| ENSRNOG00000030355 | Mroh1 | -1.04 | <0.001 | 0.010 |
| ENSRNOG00000030530 | Gzmm | 1.30 | <0.001 | 0.019 |
| ENSRNOG00000031163 | Nfkbiz | 1.23 | 0.002 | 0.036 |
| ENSRNOG00000031312 | Tnfrsf1a | 1.01 | <0.001 | 0.009 |
| ENSRNOG00000031506 | LOC120102993 | 1.05 | <0.001 | 0.002 |
| ENSRNOG00000031930 | Cela1 | 1.39 | <0.001 | 0.003 |
| ENSRNOG00000031930 | Bin2 | 1.39 | <0.001 | 0.003 |
| ENSRNOG00000032659 | Plcl1 | -1.73 | 0.001 | 0.025 |
| ENSRNOG00000032708 | RT1-Bb | 1.23 | <0.001 | <0.001 |
| ENSRNOG00000032844 | RT1-Da | 1.08 | <0.001 | 0.012 |
| ENSRNOG00000033215 | RT1-Db1 | 1.03 | <0.001 | 0.007 |
| ENSRNOG00000033256 | LOC691141 | 2.82 | <0.001 | 0.019 |
| ENSRNOG00000033433 | Csrnp1 | 1.13 | 0.003 | 0.043 |
| ENSRNOG00000033693 | Naip6 | 1.25 | 0.004 | 0.050 |
| ENSRNOG00000033932 | - | -1.14 | <0.001 | 0.021 |
| ENSRNOG00000036711 | Spn | 1.08 | <0.001 | <0.001 |
| ENSRNOG00000037298 | C1h19orf81 | -1.05 | 0.003 | 0.045 |
| ENSRNOG00000037563 | Cd68 | 1.69 | <0.001 | 0.004 |
| ENSRNOG00000038047 | - | 1.23 | <0.001 | 0.003 |
| ENSRNOG00000038881 | Hcls1 | 1.20 | <0.001 | 0.001 |
| ENSRNOG00000039390 | Slc37a2 | 1.15 | <0.001 | 0.003 |
| ENSRNOG00000039668 | Col8a1 | 1.30 | 0.002 | 0.031 |
| ENSRNOG00000039754 | Rab7b | 1.83 | 0.001 | 0.025 |
| ENSRNOG00000040198 | - | -1.13 | 0.003 | 0.042 |
| ENSRNOG00000040287 | Cyp1b1 | 1.95 | <0.001 | <0.001 |
| ENSRNOG00000042018 | RGD1306750 | -1.21 | 0.002 | 0.031 |
| ENSRNOG00000042139 | Clec4a1 | 1.34 | <0.001 | 0.004 |
| ENSRNOG00000042455 | Tlr12 | 3.00 | <0.001 | 0.004 |
| ENSRNOG00000042717 | Ciart | 3.17 | <0.001 | 0.016 |
| ENSRNOG00000042785 | Sesn2 | 1.84 | <0.001 | 0.001 |
| ENSRNOG00000042838 | - | 1.77 | <0.001 | 0.002 |
| ENSRNOG00000042897 | Nmrk2 | 1.27 | <0.001 | 0.005 |
| ENSRNOG00000043098 | Mt2A | 2.79 | <0.001 | 0.005 |
| ENSRNOG00000043416 | Bcl3 | 1.48 | <0.001 | 0.019 |
| ENSRNOG00000043451 | Spp1 | 2.72 | 0.002 | 0.032 |
| ENSRNOG00000045829 | Thbs1 | 3.17 | <0.001 | 0.005 |
| ENSRNOG00000046254 | Adgre1 | 1.05 | <0.001 | 0.006 |
| ENSRNOG00000046313 | - | 1.30 | <0.001 | 0.002 |
| ENSRNOG00000046452 | Fcgr2b | 2.18 | 0.001 | 0.027 |
| ENSRNOG00000046699 | Slpi | 2.39 | <0.001 | <0.001 |
| ENSRNOG00000047143 | Fkbp1b | 3.08 | 0.002 | 0.036 |
| ENSRNOG00000047606 | Bcl2a1 | 1.57 | <0.001 | 0.003 |
| ENSRNOG00000047800 | - | 1.06 | 0.001 | 0.021 |
| ENSRNOG00000048053 | Slfn1 | 1.55 | 0.002 | 0.034 |
| ENSRNOG00000048114 | Echdc3 | -1.11 | 0.003 | 0.038 |
| ENSRNOG00000048831 | - | 1.34 | 0.002 | 0.033 |
| ENSRNOG00000048924 | Islr | 1.27 | <0.001 | 0.019 |
| ENSRNOG00000049828 | Crlf2 | 1.12 | <0.001 | 0.013 |
| ENSRNOG00000050251 | - | 2.20 | <0.001 | <0.001 |
| ENSRNOG00000050624 | Lysmd4 | -1.32 | 0.004 | 0.048 |
| ENSRNOG00000050647 | Hspa1a | 1.49 | 0.003 | 0.044 |
| ENSRNOG00000050697 | Ctsz | 1.44 | <0.001 | 0.002 |
| ENSRNOG00000050792 | Tnfaip6 | 3.48 | <0.001 | 0.002 |
| ENSRNOG00000050819 | Birc5 | 1.49 | 0.003 | 0.04 |
| ENSRNOG00000050869 | Cebpd | 1.27 | <0.001 | 0.006 |
| ENSRNOG00000051470 | Card9 | -1.49 | <0.001 | 0.002 |
| ENSRNOG00000051690 | Clec9a | 1.26 | <0.001 | 0.014 |
| ENSRNOG00000052017 | Dio3 | 4.43 | <0.001 | 0.003 |
| ENSRNOG00000052064 | Parvg | 2.70 | 0.002 | 0.029 |
| ENSRNOG00000052070 | Aldh1a3 | 1.82 | <0.001 | 0.007 |
| ENSRNOG00000052150 | - | 3.94 | <0.001 | 0.002 |
| ENSRNOG00000052219 | Gm2a | 1.08 | <0.001 | 0.002 |
| ENSRNOG00000052389 | - | -1.14 | <0.001 | 0.010 |
| ENSRNOG00000053272 | Chi3l1 | 2.04 | <0.001 | <0.001 |
| ENSRNOG00000054212 | Pde1a | 1.10 | <0.001 | 0.006 |
| ENSRNOG00000054251 | Clec7a | 1.78 | <0.001 | 0.008 |
| ENSRNOG00000054461 | - | 1.18 | 0.003 | 0.044 |
| ENSRNOG00000054622 | - | 1.53 | 0.004 | 0.050 |
| ENSRNOG00000054957 | Sfrp4 | 3.21 | <0.001 | 0.011 |
| ENSRNOG00000055411 | - | 1.05 | 0.002 | 0.034 |
| ENSRNOG00000055650 | Pou2f2 | 1.13 | <0.001 | 0.016 |
| ENSRNOG00000055962 | Bgn | 1.62 | <0.001 | 0.002 |
| ENSRNOG00000056219 | Olr1 | 3.78 | 0.001 | 0.024 |
| ENSRNOG00000056756 | Actn1 | 1.09 | <0.001 | 0.003 |
| ENSRNOG00000057092 | - | 1.33 | <0.001 | 0.003 |
| ENSRNOG00000057501 | Fam81a | -2.03 | <0.001 | 0.002 |
| ENSRNOG00000057855 | F5 | 2.14 | 0.002 | 0.033 |
| ENSRNOG00000058003 | Spon1 | 1.41 | <0.001 | 0.007 |
| ENSRNOG00000058186 | Errfi1 | 1.38 | 0.001 | 0.026 |
| ENSRNOG00000058388 | Zfp36 | 1.19 | <0.001 | 0.017 |
| ENSRNOG00000058545 | Arhgap4 | 1.31 | <0.001 | 0.021 |
| ENSRNOG00000058645 | Tnc | 4.64 | 0.001 | 0.022 |
| ENSRNOG00000059857 | Rnd1 | 2.23 | <0.001 | 0.005 |
| ENSRNOG00000059947 | Sdc1 | 2.64 | <0.001 | 0.003 |
| ENSRNOG00000060057 | - | -1.46 | <0.001 | 0.001 |
| ENSRNOG00000061379 | - | 1.50 | 0.001 | 0.021 |
| ENSRNOG00000061850 | - | -1.09 | <0.001 | 0.013 |
| ENSRNOG00000062252 | - | 1.30 | <0.001 | <0.001 |

# Supplemental Table 4. Gene set enrichment analysis: Gene Ontology top activated and suppressed biological processes in rat heart with 40 mg/kg BW/d 2-MCPD exposure.

1. Top activated:

| ID | Description | Enrichment Score | FDR |
| --- | --- | --- | --- |
| GO:0006954 | inflammatory response | 0.545 | 3.63E-29 |
| GO:0001819 | positive regulation of cytokine production | 0.562 | 5.71E-22 |
| GO:0002252 | immune effector process | 0.529 | 6.53E-21 |
| GO:0046649 | lymphocyte activation | 0.481 | 2.29E-19 |
| GO:0050900 | leukocyte migration | 0.578 | 9.00E-19 |
| GO:0040017 | positive regulation of locomotion | 0.488 | 2.75E-17 |
| GO:0050865 | regulation of cell activation | 0.493 | 2.77E-17 |
| GO:2000147 | positive regulation of cell motility | 0.488 | 4.19E-17 |
| GO:0009617 | response to bacterium | 0.476 | 4.34E-17 |
| GO:0050778 | positive regulation of immune response | 0.488 | 5.13E-17 |
| GO:0002443 | leukocyte mediated immunity | 0.559 | 1.65E-16 |
| GO:0030335 | positive regulation of cell migration | 0.485 | 2.11E-16 |
| GO:0002253 | activation of immune response | 0.519 | 3.09E-16 |
| GO:0002694 | regulation of leukocyte activation | 0.492 | 6.05E-16 |
| GO:0060326 | cell chemotaxis | 0.581 | 7.90E-16 |
| GO:0042330 | taxis | 0.538 | 2.11E-15 |
| GO:0002250 | adaptive immune response | 0.543 | 4.76E-15 |
| GO:0006935 | chemotaxis | 0.537 | 4.79E-15 |
| GO:0042110 | T cell activation | 0.496 | 5.14E-15 |
| GO:0002683 | negative regulation of immune system process | 0.506 | 6.76E-15 |
| GO:0002263 | cell activation involved in immune response | 0.578 | 1.10E-14 |
| GO:0002757 | immune response-activating signaling pathway | 0.521 | 1.29E-14 |
| GO:0045785 | positive regulation of cell adhesion | 0.498 | 1.53E-14 |
| GO:0002521 | leukocyte differentiation | 0.474 | 1.81E-14 |
| GO:0002764 | immune response-regulating signaling pathway | 0.518 | 1.81E-14 |

1. Top Suppressed:

| ID | Description | Enrichment Score | FDR |
| --- | --- | --- | --- |
| GO:0006091 | generation of precursor metabolites and energy | -0.554 | 1.43E-29 |
| GO:0015980 | energy derivation by oxidation of organic compounds | -0.598 | 9.80E-28 |
| GO:0045333 | cellular respiration | -0.632 | 4.66E-25 |
| GO:0009060 | aerobic respiration | -0.665 | 8.32E-24 |
| GO:0006119 | oxidative phosphorylation | -0.701 | 1.64E-20 |
| GO:0022904 | respiratory electron transport chain | -0.728 | 2.29E-19 |
| GO:0022900 | electron transport chain | -0.675 | 4.19E-17 |
| GO:0042773 | ATP synthesis coupled electron transport | -0.749 | 4.34E-17 |
| GO:0042775 | mitochondrial ATP synthesis coupled electron transport | -0.746 | 3.93E-16 |
| GO:0033108 | mitochondrial respiratory chain complex assembly | -0.701 | 1.35E-15 |
| GO:0019646 | aerobic electron transport chain | -0.762 | 3.87E-15 |
| GO:0010257 | NADH dehydrogenase complex assembly | -0.738 | 3.86E-12 |
| GO:0032981 | mitochondrial respiratory chain complex I assembly | -0.738 | 3.86E-12 |
| GO:0060047 | heart contraction | -0.524 | 3.86E-12 |
| GO:0008016 | regulation of heart contraction | -0.552 | 2.08E-11 |
| GO:0060048 | cardiac muscle contraction | -0.576 | 1.74E-10 |
| GO:0016054 | organic acid catabolic process | -0.511 | 1.84E-10 |
| GO:0046395 | carboxylic acid catabolic process | -0.511 | 1.84E-10 |
| GO:0006941 | striated muscle contraction | -0.536 | 1.95E-10 |
| GO:0044282 | small molecule catabolic process | -0.458 | 3.74E-10 |
| GO:0003015 | heart process | -0.485 | 1.20E-09 |
| GO:1902600 | proton transmembrane transport | -0.607 | 1.53E-09 |
| GO:0007005 | mitochondrion organization | -0.364 | 7.83E-09 |
| GO:0032787 | monocarboxylic acid metabolic process | -0.358 | 2.01E-08 |
| GO:0061337 | cardiac conduction | -0.673 | 2.46E-08 |

# Supplemental Table 5. Gene set enrichment analysis: Gene Ontology fibroblast related biological processes in rat heart with 40 mg/kg BW/d 2-MCPD exposure.

| ID | Description | Enrichment score | FDR |
| --- | --- | --- | --- |
| GO:0010762 | regulation of fibroblast migration | 0.571 | 0.004 |
| GO:0010761 | fibroblast migration | 0.484 | 0.014 |
| GO:0048146 | positive regulation of fibroblast proliferation | 0.483 | 0.021 |
| GO:0044344 | cellular response to fibroblast growth factor stimulus | 0.435 | 0.022 |
| GO:0071774 | response to fibroblast growth factor | 0.430 | 0.024 |
| GO:0010764 | negative regulation of fibroblast migration | 0.762 | 0.028 |
| GO:0048144 | fibroblast proliferation | 0.396 | 0.029 |

# Supplemental Table 6. Rat heart oxidative stress qPCR panel

| Gene | LFC | *P* value | FDR |
| --- | --- | --- | --- |
| 18S | 0.042 | 0.723 | 0.812 |
| Aass | 0.795 | 0.137 | 0.268 |
| Actb | 1.22 | <0.001 | 0.002 |
| Als2 | -0.096 | 0.721 | 0.812 |
| Apc | 0.049 | 0.472 | 0.600 |
| Apoe | 1.03 | <0.001 | 0.007 |
| Aqr | 0.322 | 0.010 | 0.044 |
| Atr | 0.625 | 0.002 | 0.014 |
| B2m | 0.357 | 0.036 | 0.118 |
| Cat | -0.040 | 0.985 | 0.985 |
| Ccs | 0.216 | 0.090 | 0.217 |
| Ctsb | 0.670 | 0.001 | 0.011 |
| Cygb | 0.735 | <0.001 | 0.004 |
| Dhcr24 | 0.054 | 0.547 | 0.673 |
| Dnm2 | 0.175 | 0.160 | 0.277 |
| Duox1 | 0.066 | 0.475 | 0.600 |
| Duox2 | 0.699 | 0.037 | 0.118 |
| Ehd2 | 0.007 | 0.280 | 0.417 |
| Ercc2 | 0.186 | 0.093 | 0.217 |
| Ercc6 | 0.289 | 0.038 | 0.118 |
| Fancc | 0.296 | 0.016 | 0.065 |
| Fmo2 | -0.423 | 0.128 | 0.264 |
| Gab1 | 0.103 | 0.072 | 0.198 |
| Gapdh | -0.093 | 0.697 | 0.809 |
| Gpx1 | 0.115 | 0.313 | 0.439 |
| Gpx2 | 1.26 | 0.049 | 0.149 |
| Gpx3 | 0.628 | 0.124 | 0.262 |
| Gpx4 | 0.097 | 0.148 | 0.275 |
| Gpx7 | 0.831 | <0.001 | 0.005 |
| Gpx8 | 0.814 | <0.001 | 0.009 |
| Gsr | 0.452 | 0.004 | 0.022 |
| Gstk1 | -0.262 | 0.086 | 0.217 |
| Gusb | 0.768 | 0.004 | 0.023 |
| Hbb;Hba1 | -0.382 | 0.146 | 0.275 |
| Hmbs | -0.012 | 0.823 | 0.881 |
| Hprt1 | 0.047 | 0.372 | 0.498 |
| Idh1 | 0.438 | 0.003 | 0.020 |
| Ift172 | 0.061 | 0.341 | 0.463 |
| Kif9 | 0.487 | 0.083 | 0.217 |
| Mb | -0.297 | 0.138 | 0.268 |
| Mpo | -0.716 | 0.643 | 0.76 |
| Mpp4 | -0.051 | 0.979 | 0.985 |
| Mss51 | 0.319 | 0.151 | 0.275 |
| Ncf1 | -0.247 | 0.154 | 0.275 |
| Ncf2 | 2.07 | 0.002 | 0.014 |
| Ngb | 1.06 | 0.139 | 0.268 |
| Nos2 | 0.951 | 0.029 | 0.101 |
| Nox4 | 0.574 | <0.001 | 0.009 |
| Noxo1 | 1.26 | 0.112 | 0.254 |
| Nqo1 | 0.304 | 0.023 | 0.083 |
| Nudt1 | 0.244 | 0.117 | 0.260 |
| Nudt15 | 0.808 | 0.053 | 0.156 |
| Nxn | 0.136 | 0.302 | 0.436 |
| Park7 | -0.071 | 0.752 | 0.835 |
| Pgk1 | -0.048 | 0.967 | 0.985 |
| Ppp1r15b | 0.524 | 0.002 | 0.014 |
| Prdx1 | 0.143 | 0.426 | 0.562 |
| Prdx2 | 0.082 | 0.199 | 0.318 |
| Prdx3 | -0.157 | 0.319 | 0.440 |
| Prdx4 | 0.764 | <0.001 | 0.004 |
| Prdx5 | -0.085 | 0.784 | 0.859 |
| Prdx6 | 0.027 | 0.508 | 0.634 |
| Prnp | 0.274 | 0.021 | 0.083 |
| Psmb5 | -0.121 | 0.445 | 0.578 |
| Ptgs1 | 0.581 | 0.005 | 0.024 |
| Ptgs2 | 3.24 | 0.002 | 0.014 |
| Rplp0 | 0.502 | 0.009 | 0.044 |
| Rplp2 | 0.008 | 0.796 | 0.862 |
| Scd1 | -1.509 | 0.274 | 0.415 |
| Slc38a1 | 0.023 | 0.585 | 0.701 |
| Slc38a4 | -0.296 | 0.314 | 0.439 |
| Slc38a5 | 0.016 | 0.864 | 0.900 |
| Slc41a3 | -0.287 | 0.175 | 0.289 |
| Sod1 | -0.068 | 0.870 | 0.900 |
| Sod2 | -0.011 | 0.702 | 0.809 |
| Sod3 | -0.243 | 0.289 | 0.424 |
| Srxn1 | 1.88 | <0.001 | 0.002 |
| Tbp | 0.204 | 0.180 | 0.292 |
| Tfrc | 0.314 | 0.084 | 0.217 |
| Tmod1 | -0.174 | 0.217 | 0.341 |
| Txnip | -0.395 | 0.164 | 0.277 |
| Txnrd1 | 1.02 | 0.002 | 0.014 |
| Txnrd2 | -0.165 | 0.577 | 0.700 |
| Ubc | 0.209 | 0.023 | 0.083 |
| Ucp3 | -0.412 | 0.057 | 0.161 |
| Vim | 0.662 | 0.011 | 0.049 |
| Xpa | -0.006 | 0.834 | 0.883 |

# Supplemental Table 7. Identified rat heart proteins expression

| Protein | Symbol | LFC | *P* value | FDR |
| --- | --- | --- | --- | --- |
| 14-3-3 protein epsilon | 1433E | 0.759 | <0.001 | 0.008 |
| 2,4-dienoyl-CoA reductase, mitochondrial | DECR | -0.496 | 0.036 | 0.179 |
| 26 s protease regulatory subunit 4 | PRS4 | -0.838 | <0.001 | 0.011 |
| 26S protease regulatory subunit 7 | PRS7 | -0.351 | 0.160 | 0.380 |
| 2-oxoisovalerate dehydrogenase subunit alpha, mitochondrial | ODBA | -0.621 | 0.005 | 0.061 |
| 3-hydroxyisobutyrate dehydrogenase, mitochondrial | 3HIDH | -0.361 | 0.022 | 0.142 |
| 3-ketoacyl-CoA thiolase, mitochondrial | THIM | 1.31 | <0.001 | 0.022 |
| 78 kDa glucose-regulated protein | GRP78 | -0.554 | 0.040 | 0.184 |
|  |  | -0.469 | 0.002 | 0.036 |
|  |  | -0.228 | 0.379 | 0.604 |
| Acetyl-CoA acetyltransferase, mitochondrial | THIL | -1.09 | <0.001 | <0.001 |
|  |  | -0.253 | 0.230 | 0.454 |
|  |  | 1.66 | <0.001 | 0.008 |
|  |  | 1.69 | 0.001 | 0.028 |
| Actin aortic smooth muscle | ACTA | -0.706 | 0.017 | 0.119 |
|  |  | -0.576 | 0.008 | 0.073 |
|  |  | -0.517 | 0.005 | 0.061 |
|  |  | -0.500 | 0.082 | 0.265 |
|  |  | -0.459 | 0.055 | 0.217 |
|  |  | -0.175 | 0.372 | 0.599 |
| Actin, alpha cardiac muscle 1 | ACTC | 0.34 | 0.572 | 0.748 |
| Acyl-CoA synthetase family member 2, mitochondrial | ACSF2 | 0.731 | <0.001 | 0.023 |
| Aldehyde dehydrogenase, mitochondrial | ALDH2 | -0.427 | 0.077 | 0.259 |
|  |  | -0.427 | 0.077 | 0.259 |
|  |  | -0.308 | 0.295 | 0.528 |
|  |  | -0.308 | 0.295 | 0.528 |
| Alpha-2-HS-glycoprotein | FETUA | -0.403 | 0.016 | 0.116 |
|  |  | -0.382 | 0.027 | 0.154 |
|  |  | -0.338 | 0.072 | 0.250 |
| Alpha-actinin-1 | ACTN1 | -1.07 | 0.005 | 0.060 |
| Alpha-enolase | ENOA | 1.18 | <0.001 | <0.001 |
| Annexin A1 | ANXA1 | -0.645 | 0.015 | 0.113 |
| Annexin A4 | ANXA4 | -0.423 | 0.006 | 0.062 |
| Annexin A5 | ANXA5 | -0.339 | 0.055 | 0.217 |
| Aspartate aminotransferase, cytoplasmic | AATC | -0.619 | 0.016 | 0.115 |
| ATP synthase subunit alpha, mitochondrial | ATPA | -0.692 | 0.043 | 0.190 |
|  |  | -0.647 | 0.025 | 0.151 |
|  |  | 0.089 | 0.837 | 0.914 |
|  |  | 0.539 | 0.003 | 0.043 |
|  |  | 1.06 | <0.001 | 0.020 |
| ATP synthase subunit beta, mitochondrial | ATPB | 0.797 | 0.001 | 0.025 |
| ATP synthase subunit gamma, mitochondrial | ATPG | -0.448 | 0.21 | 0.431 |
|  |  | -0.448 | 0.21 | 0.431 |
|  |  | -0.249 | 0.117 | 0.318 |
|  |  | -0.249 | 0.117 | 0.318 |
| Beta-enolase | ENOB | 0.968 | <0.001 | <0.001 |
| cAMP-dependent protein kinase type I-alpha regulatory subunit | KAP0 | -0.253 | 0.141 | 0.350 |
| Catalase | CATA | 0.846 | <0.001 | 0.020 |
| Ceruloplasmin | CERU | -0.488 | 0.133 | 0.342 |
| Coiled-coil domain-containing protein 22 | CCD22 | -0.632 | 0.009 | 0.080 |
| Complement C3 | CO3 | -0.300 | 0.413 | 0.635 |
|  |  | -0.282 | 0.137 | 0.345 |
| Coronin-1A | COR1A | -1.01 | <0.001 | <0.001 |
| Cytoglobin | CYGB | -0.507 | 0.039 | 0.183 |
| Cytoplasmic aconitate hydratase | ACOC | -0.705 | 0.023 | 0.144 |
| Cytosol aminopeptidase | AMPL | -0.388 | 0.040 | 0.184 |
| Cytosolic non-specific dipeptidase | CNDP2 | -0.329 | 0.046 | 0.196 |
| Delta(3,5)-Delta(2,4)-dienoyl-CoA isomerase, mitochondrial | ECH1 | -0.969 | 0.005 | 0.059 |
| Desmin | DESM | -0.361 | 0.331 | 0.561 |
| Dihydrolipoyl dehydrogenase, mitochondrial | DLDH | -1.40 | <0.001 | <0.001 |
| Dihydrolipoyllysine-residue acetyltransferase component of pyruvate dehydrogenase complex, mitochondrial | ODP2 | -1.02  -0.360  -0.290  1.24 | <0.001  0.015  0.244  <0.001 | 0.009  0.113  0.474  <0.001 |
| EH domain-containing protein 1 | EHD1 | -0.298 | 0.069 | 0.243 |
| Electron transfer flavoprotein subunit beta | ETFB | 0.122 | 0.624 | 0.780 |
|  |  | 0.635 | <0.001 | 0.021 |
| Endoplasmic reticulum resident protein 29 | ERP29 | -0.447 | 0.007 | 0.073 |
| Endoplasmin | ENPL | -0.51 | 0.007 | 0.073 |
| Enoyl-CoA delta isomerase 1, mitochondrial | ECI1 | -0.667 | 0.010 | 0.087 |
| Ethanolamine-phosphate cytidylyltransferase | PCY2 | 0.653 | <0.001 | 0.014 |
| Eukaryotic initiation factor 4A-II | IF4A2 | -0.881 | 0.019 | 0.130 |
| Eukaryotic translation initiation factor 2 subunit 1 | IF2A | -0.344 | 0.075 | 0.257 |
| Eukaryotic translation initiation factor 3 subunit I | EIF3I | -0.371 | 0.152 | 0.370 |
| Evolutionarily conserved signaling intermediate in Toll pathway, mitochondrial | ECSIT | 0.677 | <0.001 | 0.012 |
| F-actin-capping protein subunit beta | CAPZB | -0.425 | 0.012 | 0.095 |
| Ferritin light chain 1 | FRIL1 | -0.572 | <0.001 | 0.019 |
|  |  | -0.572 | <0.001 | 0.019 |
| Four and a half LIM domains protein 2 | FHL2 | 0.883 | <0.001 | 0.003 |
| Fructose-bisphosphate aldolase A | ALDOA | -0.589 | 0.162 | 0.381 |
| Fumarate hydratase, mitochondrial | FUMH | 0.495 | 0.004 | 0.047 |
| Gelsolin | GELS | -0.694 | 0.009 | 0.079 |
|  |  | -0.525 | 0.015 | 0.113 |
|  |  | -0.383 | 0.067 | 0.239 |
| Glutamate receptor 3 | GRIA3 | -0.927 | <0.001 | 0.020 |
| Glutathione peroxidase 1 | GPX1 | 0.486 | 0.212 | 0.432 |
| Glycerin-3-phosphate dehydrogenase [NAD+], cytoplasmic | GPDA | 0.665 | 0.141 | 0.350 |
| Glycogen phosphorylase, muscle form | PYGM | -0.219 | 0.231 | 0.456 |
| GrpE protein homolog 1, mitochondrial | GRPE1 | -0.398 | 0.041 | 0.184 |
| Guanine deaminase | GUAD | -0.832 | <0.001 | 0.002 |
|  |  | -0.638 | 0.047 | 0.197 |
| Guanine nucleotide-binding protein subunit beta-2-like 1 | GBLP | -0.609 | 0.003 | 0.041 |
| Haptoglobin | HPT | -0.509 | 0.067 | 0.239 |
|  |  | -0.377 | 0.175 | 0.396 |
| Heat shock 70 kDa protein 4 | HSP74 | -0.459 | 0.011 | 0.093 |
|  |  | -0.293 | 0.150 | 0.368 |
| Heat shock protein beta-1 | HSPB1 | -0.424 | <0.001 | 0.020 |
| Heat shock protein HSP 90-beta | HS90B | -0.543 | 0.025 | 0.150 |
|  |  | -0.459 | 0.033 | 0.169 |
|  |  | -0.239 | 0.122 | 0.324 |
| Hemopexin | HEMO | -0.521 | 0.010 | 0.085 |
|  |  | -0.442 | 0.029 | 0.161 |
| Heterogeneous nuclear ribonucleoprotein F | HNRPF | -0.581 | 0.004 | 0.053 |
| Hydroxyacyl-coenzyme A dehydrogenase, mitochondrial | HCDH | 0.498 | 0.003 | 0.043 |
| Kinesin heavy chain isoform 5A | KIF5A | 0.507 | 0.008 | 0.073 |
| Lamin-B1 | LMNB1 | -0.508 | 0.015 | 0.113 |
|  |  | -0.467 | 0.008 | 0.079 |
| L-lactate dehydrogenase B chain | LDHB | -0.628 | 0.057 | 0.220 |
|  |  | -0.427 | 0.130 | 0.337 |
| Macrophage-capping protein | CAPG | -0.759 | <0.001 | 0.006 |
| Malate dehydrogenase, mitochondrial | MDHM | 0.156 | 0.437 | 0.650 |
|  |  | 0.533 | 0.041 | 0.186 |
| Methylmalonate-semialdehyde dehydrogenase [acylating], mitochondrial | MMSA | -0.453  0.501  0.931 | 0.015  0.005  <0.001 | 0.113  0.061  0.022 |
| Mitochondrial inner membrane protein (Fragment) | MIC60 | -0.387 | 0.099 | 0.290 |
| Mu-crystallin homolog | CRYM | -0.732 | <0.001 | 0.003 |
| Murinoglobulin-1 | MUG1 | -0.416 | 0.076 | 0.258 |
| Myosin light chain 3 | MYL3 | -0.162 | 0.379 | 0.604 |
|  |  | 0.404 | 0.058 | 0.221 |
| Myosin regulatory light chain 2, ventricular/cardiac muscle isoform | MLRV | 1.10 | 0.016 | 0.115 |
| Myosin-6 | MYH6 | -0.793 | 0.025 | 0.150 |
|  |  | -0.714 | 0.029 | 0.161 |
|  |  | -0.686 | 0.003 | 0.044 |
|  |  | -0.649 | 0.095 | 0.282 |
|  |  | -0.634 | 0.073 | 0.252 |
| Myosin-6 (continued) | MYH6 | -0.591 | 0.031 | 0.165 |
|  |  | -0.520 | 0.036 | 0.177 |
|  |  | 0.366 | 0.268 | 0.502 |
| Myosin-7 | MYH7 | -0.789 | 0.013 | 0.103 |
| Myosin-binding protein C, cardiac-type | MYPC | -2.09 | <0.001 | <0.001 |
|  |  | -0.690 | 0.008 | 0.074 |
|  |  | -0.651 | 0.036 | 0.178 |
|  |  | -0.432 | 0.038 | 0.181 |
| N(G),N(G)-dimethylarginine dimethylaminohydrolase 2 | DDAH2 | -0.358 | 0.029 | 0.161 |
| Nck-associated protein 1 | NCKP1 | -1.35 | <0.001 | <0.001 |
| Plasminogen | PLMN | -0.555 | 0.011 | 0.093 |
|  |  | -0.410 | 0.044 | 0.193 |
|  |  | -0.369 | 0.093 | 0.279 |
| Pre-mRNA-processing factor 19 | PRP19 | -0.122 | 0.299 | 0.533 |
| Protein disulfide-isomerase A3 | PDIA3 | -0.613 | 0.009 | 0.079 |
|  |  | -0.563 | 0.001 | 0.026 |
|  |  | -0.449 | 0.001 | 0.024 |
|  |  | -0.241 | 0.449 | 0.659 |
| Protein DJ-1 | PARK7 | 1.27 | <0.001 | <0.001 |
| Purine nucleoside phosphorylase | PNPH | -0.502 | 0.001 | 0.028 |
| Pyruvate carboxylase, mitochondrial | PYC | 0.521 | 0.057 | 0.221 |
| Pyruvate dehydrogenase E1 component subunit alpha, somatic form, mitochondrial | ODPA | 0.447  0.718  0.757 | 0.206  0.046  0.004 | 0.430  0.196  0.050 |
| Pyruvate kinase isozymes M1/M2 | PKM1/M2 | -0.266 | 0.229 | 0.454 |
| Sarcolemmal membrane-associated protein | SLMAP | -0.390 | 0.024 | 0.150 |
| Sarcosine dehydrogenase, mitochondrial | SARDH | -0.312 | 0.032 | 0.166 |
| Septin-8 | SEPT8 | -0.445 | 0.076 | 0.258 |
| Serine protease inhibitor A3N | SPA3N | -0.484 | 0.002 | 0.034 |
|  |  | -0.456 | 0.002 | 0.030 |
| Serine/threonine-protein phosphatase 2A 55 kDa regulatory subunit B alpha isoform | 2ABA | -0.438 | 0.065 | 0.235 |
| Serine--pyruvate aminotransferase, mitochondrial | SPYA | 0.136 | 0.575 | 0.748 |
| Transferrin | TRFE | -0.714 | 0.005 | 0.058 |
|  |  | -0.454 | 0.014 | 0.109 |
|  |  | -0.349 | 0.389 | 0.613 |
|  |  | 0.364 | 0.165 | 0.381 |
| Serum albumin | ALBU | -1.51 | <0.001 | 0.002 |
|  |  | -1.21 | <0.001 | 0.022 |
|  |  | -0.851 | <0.001 | 0.008 |
|  |  | -0.828 | <0.001 | 0.010 |
|  |  | -0.819 | 0.002 | 0.035 |
|  |  | -0.695 | <0.001 | 0.022 |
|  |  | -0.636 | 0.011 | 0.093 |
| Serum albumin (continued) | ALBU | -0.627 | 0.001 | 0.026 |
|  |  | -0.565 | <0.001 | 0.020 |
|  |  | -0.525 | 0.190 | 0.411 |
|  |  | -0.446 | 0.040 | 0.184 |
|  |  | -0.421 | 0.163 | 0.381 |
|  |  | 0.024 | 0.902 | 0.949 |
|  |  | 0.296 | 0.289 | 0.523 |
|  |  | 0.900 | <0.001 | 0.007 |
| Succinyl-CoA:3-ketoacid coenzyme A transferase 1, mitochondrial | SCOT1 | -0.717  -0.585  -0.353  -0.321 | 0.044  <0.001  0.046  0.047 | 0.192  0.011  0.197  0.198 |
| Thioredoxin reductase 2, mitochondrial | TRXR2 | -0.767 | 0.003 | 0.037 |
|  |  | -0.751 | 0.007 | 0.071 |
| Trifunctional enzyme subunit alpha, mitochondrial | ECHA | 0.400 | 0.243 | 0.474 |
|  |  | 0.631 | 0.014 | 0.108 |
|  |  | 0.657 | <0.001 | 0.014 |
| Tripartite motif-containing protein 72 | TRI72 | -0.516 | 0.003 | 0.044 |
| Tubulin alpha-1A chain | TBA1A | -0.963 | <0.001 | 0.016 |
|  |  | -0.963 | <0.001 | 0.016 |
| Ubiquitin-like modifier-activating enzyme 1 | UBA1 | -1.24 | 0.001 | 0.028 |
| Vimentin | VIME | -1.00 | <0.001 | 0.020 |
|  |  | -0.809 | 0.002 | 0.031 |
|  |  | -0.807 | 0.025 | 0.150 |
| Vinculin | VINC | -0.992 | <0.001 | 0.008 |
|  |  | 0.73 | 0.021 | 0.138 |
| Vitamin D-binding protein | VTDB | -0.458 | 0.033 | 0.168 |
| Voltage-dependent anion-selective channel protein 1 | VDAC1 | -0.038 | 0.877 | 0.934 |
| Voltage-dependent anion-selective channel protein 2 | VDAC2 | -1.15  0.431 | <0.001  0.031 | 0.003  0.165 |

# Supplemental Table 8. Oxylipin metabolism related genes.

| ENSEMBL | Gene | LFC | *P* value | FDR |
| --- | --- | --- | --- | --- |
| ENSRNOG00000027037 | Alox12 | 0.186 | 0.853 | 0.944 |
| ENSRNOG00000019183 | Alox15 | 0.210 | 0.636 | 0.848 |
| ENSRNOG00000012972 | Alox5 | -0.193 | 0.526 | 0.785 |
| ENSRNOG00000049911 | Cbr1 | -0.062 | 0.815 | 0.928 |
| ENSRNOG00000049911 | Cbr1l2 | -0.062 | 0.815 | 0.928 |
| ENSRNOG00000001701 | Cbr3 | 0.466 | 0.053 | 0.240 |
| ENSRNOG00000040287 | Cyp1b1 | 1.95 | <0.001 | <0.001 |
| ENSRNOG00000012458 | Cyp2e1 | -1.77 | <0.001 | 0.002 |
| ENSRNOG00000031004 | Cyp2j3 | 0.183 | 0.725 | 0.890 |
| ENSRNOG00000011053 | Cyp2u1 | -0.147 | 0.545 | 0.795 |
| ENSRNOG00000055078 | Cyp4b1 | -0.918 | <0.001 | 0.010 |
| ENSRNOG00000004786 | Cyp4f1 | -0.131 | 0.795 | 0.919 |
| ENSRNOG00000043233 | Cyp4f6 | -0.154 | 0.431 | 0.724 |
| ENSRNOG00000018239 | Dhrs4 | -0.419 | 0.005 | 0.057 |
| ENSRNOG00000003515 | Ephx1 | 0.044 | 0.857 | 0.946 |
| ENSRNOG00000017286 | Ephx2 | -0.528 | 0.012 | 0.104 |
| ENSRNOG00000062276 | Ggt5 | 0.506 | 0.043 | 0.217 |
| ENSRNOG00000048812 | Gpx1 | 0.092 | 0.573 | 0.812 |
| ENSRNOG00000052564 | Gpx3 | 0.346 | 0.290 | 0.607 |
| ENSRNOG00000009751 | Gpx7 | 0.393 | 0.068 | 0.279 |
| ENSRNOG00000010461 | Gpx8 | 0.928 | <0.001 | <0.001 |
| ENSRNOG00000019221 | Gstm4 | -0.117 | 0.636 | 0.848 |
| ENSRNOG00000004494 | Lta4h | 0.006 | 0.983 | 0.992 |
| ENSRNOG00000003244 | Ltc4s | -0.961 | 0.014 | 0.111 |
| ENSRNOG00000061857 | Mgst2 | -0.471 | 0.050 | 0.233 |
| ENSRNOG00000004245 | Mgst3 | -0.561 | 0.085 | 0.317 |
| ENSRNOG00000057470 | Pla2g12a | -0.261 | 0.479 | 0.757 |
| ENSRNOG00000016945 | Pla2g2a | -0.759 | 0.019 | 0.135 |
| ENSRNOG00000016826 | Pla2g2d | 0.760 | 0.006 | 0.068 |
| ENSRNOG00000007447 | Pla2g4b | -0.213 | 0.564 | 0.807 |
| ENSRNOG00000016838 | Pla2g5 | -0.959 | 0.002 | 0.037 |
| ENSRNOG00000012295 | Pla2g6 | -0.200 | 0.526 | 0.785 |
| ENSRNOG00000015550 | Ptgds | -0.213 | 0.460 | 0.745 |
| ENSRNOG00000014050 | Ptges2 | -0.04 | 0.855 | 0.945 |
| ENSRNOG00000002642 | Ptges3 | 0.107 | 0.606 | 0.832 |
| ENSRNOG00000060486 | Ptges3l1 | 0.436 | 0.043 | 0.215 |
| ENSRNOG00000008245 | Ptgis | 0.737 | 0.014 | 0.112 |
| ENSRNOG00000007415 | Ptgs1 | 0.496 | 0.043 | 0.217 |
| ENSRNOG00000002525 | Ptgs2 | 2.37 | 0.007 | 0.071 |
| ENSRNOG00000007918 | Tbxas1 | 0.761 | 0.040 | 0.208 |

# Supplemental Table 9. Rat heart oxylipin expression with 40 mg/kg BW/d 2-MCPD exposure.

|  |  |  |  |  |  |  |
| --- | --- | --- | --- | --- | --- | --- |
| Oxylipins | Enz | LFC | *P* value | FDR |  |  |
| LA oxylipins |  |  |  |  |  |  |
| 9,10-EpOME | CYPe | -0.112 | 0.805 | 0.979 |  |  |
| 9,10-DiHOME | CYPe | -0.030 | 0.762 | 0.979 |  |  |
| 12,13-EpOME | CYPe | -0.176 | 0.755 | 0.979 |  |  |
| 12,13-DiHOME | CYPe | 0.070 | 0.519 | 0.979 |  |  |
| LA-CYPe total |  | -0.108 | 0.965 | 0.965 |  |  |
|  |  |  |  |  |  |  |
| 9-HODE | LOX | -0.368 | 0.952 | 0.979 |  |  |
| 13-HODE | LOX | -0.353 | 0.979 | 0.979 |  |  |
| 13-oxoODE | LOX | -0.290 | 0.589 | 0.979 |  |  |
| (9,10,13- & 9,12,13-) triHOME | LOX | -0.402 | 0.421 | 0.979 |  |  |
| LA-LOX total |  | -0.355 | 0.934 | 0.965 |  |  |
|  |  |  |  |  |  |  |
| LA total |  | -0.353 | 0.935 | 0.935 |  |  |
|  |  |  |  |  |  |  |
| GLA oxylipins |  |  |  |  |  |  |
| 13-HOTrE-γ | LOX | -0.036 | 0.891 | 0.979 |  |  |
|  |  |  |  |  |  |  |
| DGLA oxylipins |  |  |  |  |  |  |
| TXB1 | COX | -0.216 | 0.669 | 0.979 |  |  |
| 15k-PGE1 | COX | 0.359 | 0.240 | 0.757 |  |  |
| DGLA-COX total |  | -0.066 | 0.880 | 0.979 |  |  |
|  |  |  |  |  |  |  |
| 15-HETrE | LOX | -0.059 | 0.884 | 0.979 |  |  |
|  |  |  |  |  |  |  |
| DGLA total |  | -0.059 | 0.884 | 0.979 |  |  |
|  |  |  |  |  |  |  |
| AA oxylipins |  |  |  |  |  |  |
| PGD2 | COX | -0.189 | 0.965 | 0.979 |  |  |
| 15d-PGD2 | COX | -0.341 | 0.958 | 0.979 |  |  |
| PGE2 | COX | -0.009 | 0.837 | 0.979 |  |  |
| 11b-PGE2 | COX | 0.198 | 0.730 | 0.979 |  |  |
| 15k-PGE2 | COX | -0.036 | 0.939 | 0.979 |  |  |
| 6k-PGF1a | COX | 0.624 | 0.748 | 0.979 |  |  |
| 12-HHTrE | COX | -0.266 | 0.023 | 0.203 |  |  |
| TXB2 | COX | -0.022 | 0.909 | 0.979 |  |  |
| AA-COX total |  | 0.115 | 0.611 | 0.965 |  |  |
|  |  |  |  |  |  |  |
| 16-HETE | CYPh | -0.219 | 0.172 | 0.757 |  |  |
| 20-COOH-AA | CYPh | 0.780 | 0.568 | 0.979 |  |  |
| AA-CYPh total |  | 0.494 | 0.900 | 0.965 |  |  |
|  |  |  |  |  |  |  |
| 5,6-EpETrE | CYPe | -0.700 | 0.623 | 0.979 |  |  |
| 5,6-DiHETrE | CYPe | 0.277 | 0.463 | 0.979 |  |  |
| 8,9-EpETrE | CYPe | 0.330 | 0.009 | 0.181 |  |  |
| 8,9-DiHETrE | CYPe | 0.268 | 0.245 | 0.757 |  |  |
| 11,12-EpETrE | CYPe | 0.199 | 0.471 | 0.979 |  |  |
| 11,12-DiHETrE | CYPe | 0.033 | 0.791 | 0.979 |  |  |
| 14,15-EpETrE | CYPe | -0.201 | 0.415 | 0.979 |  |  |
| 14,15-DiHETrE | CYPe | 0.180 | 0.557 | 0.979 |  |  |
| AA-CYPe |  | 0.121 | 0.275 | 0.824 |  |  |
|  |  |  |  |  |  |  |
| 5-HETE | LOX | -0.539 | 0.158 | 0.757 |  |  |
| 5-oxoETE | LOX | -1.00 | 0.187 | 0.757 |  |  |
| 8-HETE | LOX | -0.420 | 0.302 | 0.800 |  |  |
| 9-HETE | LOX | -0.361 | 0.222 | 0.757 |  |  |
| 11-HETE | LOX | -0.759 | 0.257 | 0.757 |  |  |
| 12-HETE | LOX | -0.503 | 0.275 | 0.766 |  |  |
| 12-oxoETE | LOX | -0.142 | 0.693 | 0.979 |  |  |
| 15-HETE | LOX | -0.704 | 0.212 | 0.757 |  |  |
| 15-oxoETE | LOX | -0.591 | 0.237 | 0.757 |  |  |
| LTB4 | LOX | 0.094 | 0.847 | 0.979 |  |  |
| 12-epi-LTB4 | LOX | 0.164 | 0.752 | 0.979 |  |  |
| AA-LOX total |  | -0.510 | 0.227 | 0.824 |  |  |
|  |  |  |  |  |  |  |
| AA total |  | -0.251 | 0.394 | 0.880 |  |  |
|  |  |  |  |  |  |  |
| ALA oxylipins |  |  |  |  |  |  |
| 9,10-EpODE | CYPe | -0.378 | 0.171 | 0.757 |  |  |
| 9,10-DiHODE | CYPe | -0.367 | 0.933 | 0.979 |  |  |
| 12,13-EpODE | CYPe | -0.039 | 0.903 | 0.979 |  |  |
|  |  |  |  |  |  |  |
| 9-HOTrE | LOX | -0.499 | 0.698 | 0.979 |  |  |
| 9-oxoOTrE | LOX | -0.706 | 0.078 | 0.520 |  |  |
| ALA-LOX total |  | -0.524 | 0.533 | 0.965 |  |  |
|  |  |  |  |  |  |  |
| ALA total |  | -0.514 | 0.528 | 0.880 |  |  |
|  |  |  |  |  |  |  |
| DHA oxylipins |  |  |  |  |  |  |
| 20-HDoHE | CYPh | -1.20 | 0.019 | 0.203 |  |  |
|  |  |  |  |  |  |  |
| 4-HDoHE | LOX | -2.19 | <0.001 | 0.011 |  |  |
| 10-HDoHE | LOX | 0.092 | 0.716 | 0.979 |  |  |
| 14-HDoHE | LOX | -0.909 | 0.014 | 0.181 |  |  |
| 16-HDoHE | LOX | -1.60 | 0.028 | 0.210 |  |  |
| 17-HDoHE | LOX | -0.806 | 0.013 | 0.181 |  |  |
| DHA-LOX total |  | -0.581 | 0.006 | 0.054 |  |  |
|  |  |  |  |  |  |  |
| DHA total |  | -0.715 | 0.002 | 0.009 |  |  |
|  |  |  |  |  |  |  |
| n-6 total |  | -0.351 | 0.915 | 0.915 |  |  |
| n-3 total |  | -0.627 | 0.012 | 0.023 |  |  |
|  |  |  |  |  |  |  |
| COX total |  | 0.109 | 0.620 | 0.909 |  |  |
| CYPh total |  | -0.183 | 0.561 | 0.909 |  |  |
| CYPe total |  | -0.065 | 0.875 | 0.909 |  |  |
| LOX total |  | -0.358 | 0.909 | 0.909 |  |  |
|  |  |  |  |  |  |  |
| Total |  | -0.352 | 0.910 | - |  |  |

# Supplemental Table 10. Rat heart oxidized phosphatidylcholine expression with 40 mg/kg BW/d 2-MCPD exposure.

| OxPC | LFC | *P* value | FDR |
| --- | --- | --- | --- |
| PGPC | -0.135 | 0.661 | 0.661 |
| PONPC | -0.275 | 0.337 | 0.421 |
| POVPC | -0.48 | 0.122 | 0.203 |
